# Supplementary material for: Genetic aberrations in iPSCs are introduced by a transient G1/S cell cycle checkpoint deficiency
Source: Nat Commun. 2020 Jan 10;11:197. doi: 10.1038/s41467-019-13830-x (PMC6954237; doi:10.1038/s41467-019-13830-x)
Supplement: Supplementary file 1 — Supplementary Information [file 41467_2019_13830_MOESM1_ESM.pdf]

Supplementary Information

Molecular basis of the genetic aberrations in iPS cells and a solution using human erythroblasts

Araki et al.

Supplementary Figures 1-23, Supplementary Tables 1-3 and Supplementary References

Supplementary Figure 1

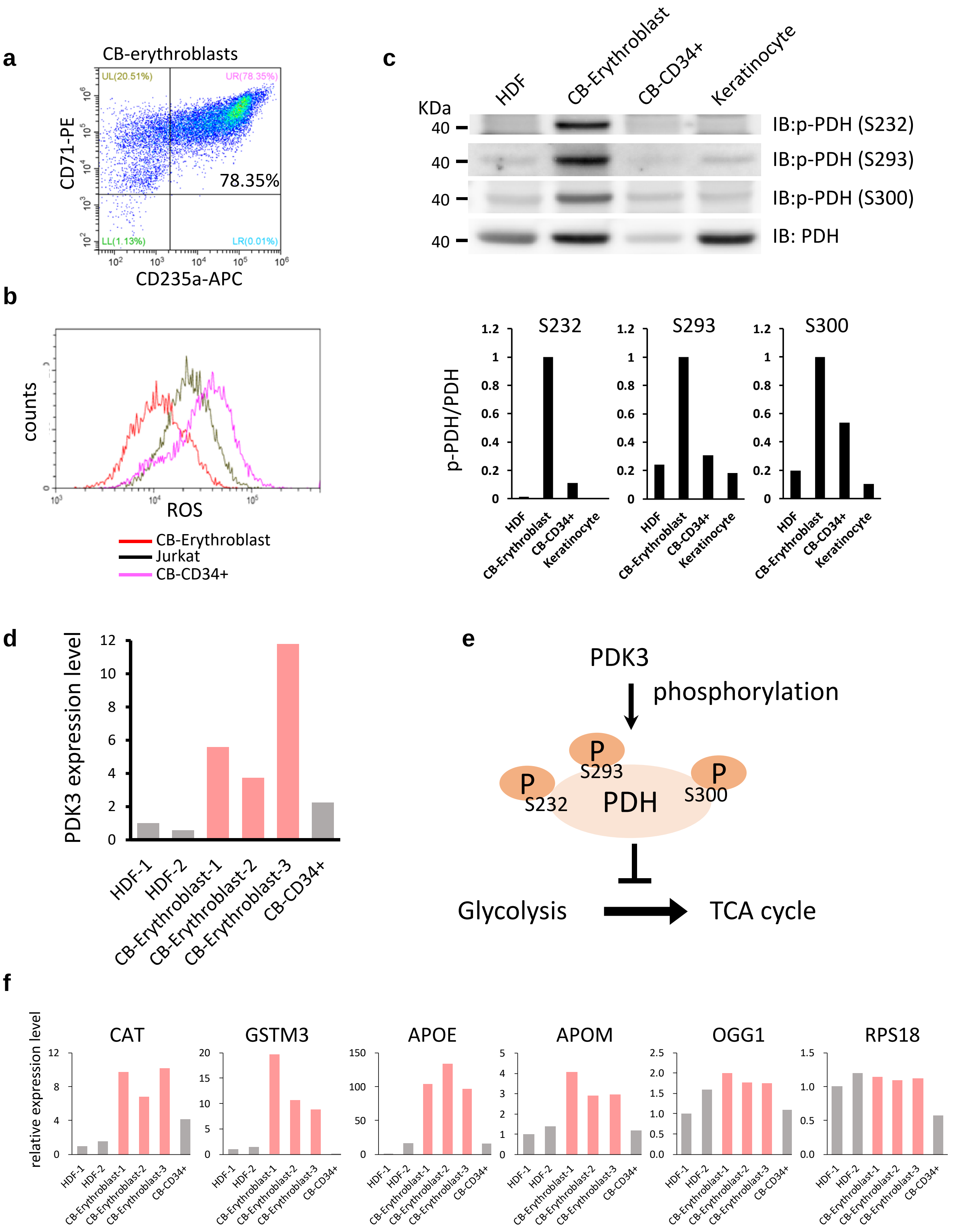

Supplementary Fig. 1 Generation of human iPSCs using erythroblasts expanded from cord blood. (**a**) Erythroblast-rich fraction expanded from cord blood (CB-erythroblasts), as demonstrated by positive staining with antibodies for CD71 and CD235a. (**b**) Cells were stained with CellROX Deep Red and analyzed by flow cytometry. (**c**) Western blot analysis of the phosphorylation of the three Ser residues in pyruvate dehydrogenase (PDH), S232, S293 and S300, which indicates the inactivation of this enzyme. (**d**) High expression of the PDK3 gene in CB-erythroblasts. The data were obtained by DNA microarray. We analyzed two independent human dermal fibroblast (HDF) cultures from two individuals, three independent erythroblast-rich fractions expanded from the cord blood of three individuals and one CD34+ -rich fraction also from cord blood. The HDF-1 value is defined as 1. (**e**) The inhibition of PDH, which catalyzes the conversion of pyruvate to acetyl-CoA and CO_2, and thereby links the glycolytic pathway to the tricarboxylic (TCA) cycle, was clearly evident in CB-erythroblasts. (**f**) Microarray analysis showing a high expression of antioxidant genes. The HDF-1 value is defined as 1. RPS18 was used as a control. Source data are provided as a Source Data file.

Supplementary Figure 2

a

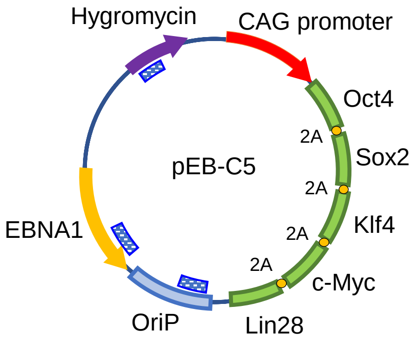

b

iPS clone	vector	bio-info	accession number**
BC1	episomal	our algorithm*	SRX115496
BCT1	episomal	“	SRX110670
E1	episomal	“	SRX110668
E2	episomal	“	SRR1022671
M1	mRNA	“	SRR3068630
M2	mRNA	“	SRR3068631
R1	retrovirus	“	SRR3068623
R2	retrovirus	“	SRR3068624
R3	retrovirus	“	SRR3068625
S1	Sendai virus	“	SRR3068627
S2	Sendai virus	“	SRR3068628
S3	Sendai virus	“	SRR3068629
HDF series	retrovirus	“	this study
CB-epi series	episomal	“	this study

*See ‘method’.
Of note, SNV identification was performed using an identical algorithm for all samples.
**the WGS data that have been reported so far by other groups were downloaded from public database and re-analyzed.

c

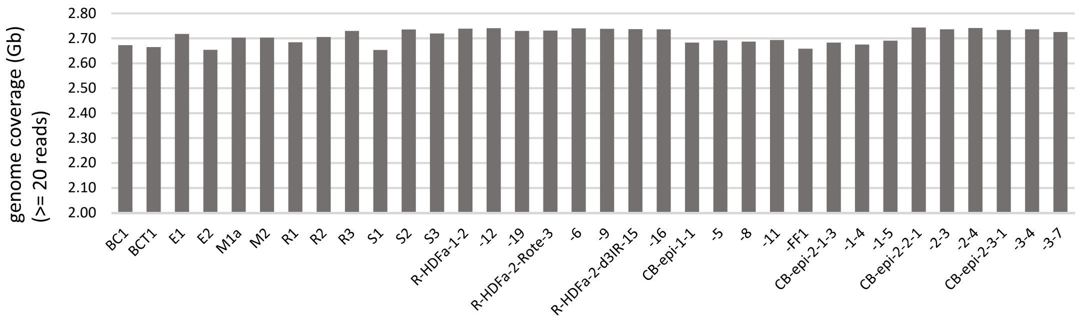

Supplementary Fig. 2 Generation of human iPSCs using erythroblasts expanded from cord blood. (**a**) The plasmid used for generating integration-free iPSCs[1] and the positions of the PCR amplicons for evaluating a genome integration-free status are indicated (blue boxes). (**b**) Human iPSC lines used for the comparison of point mutations. (**c**) A genome coverage depth >=20 is shown.

Supplementary Figure 3

a

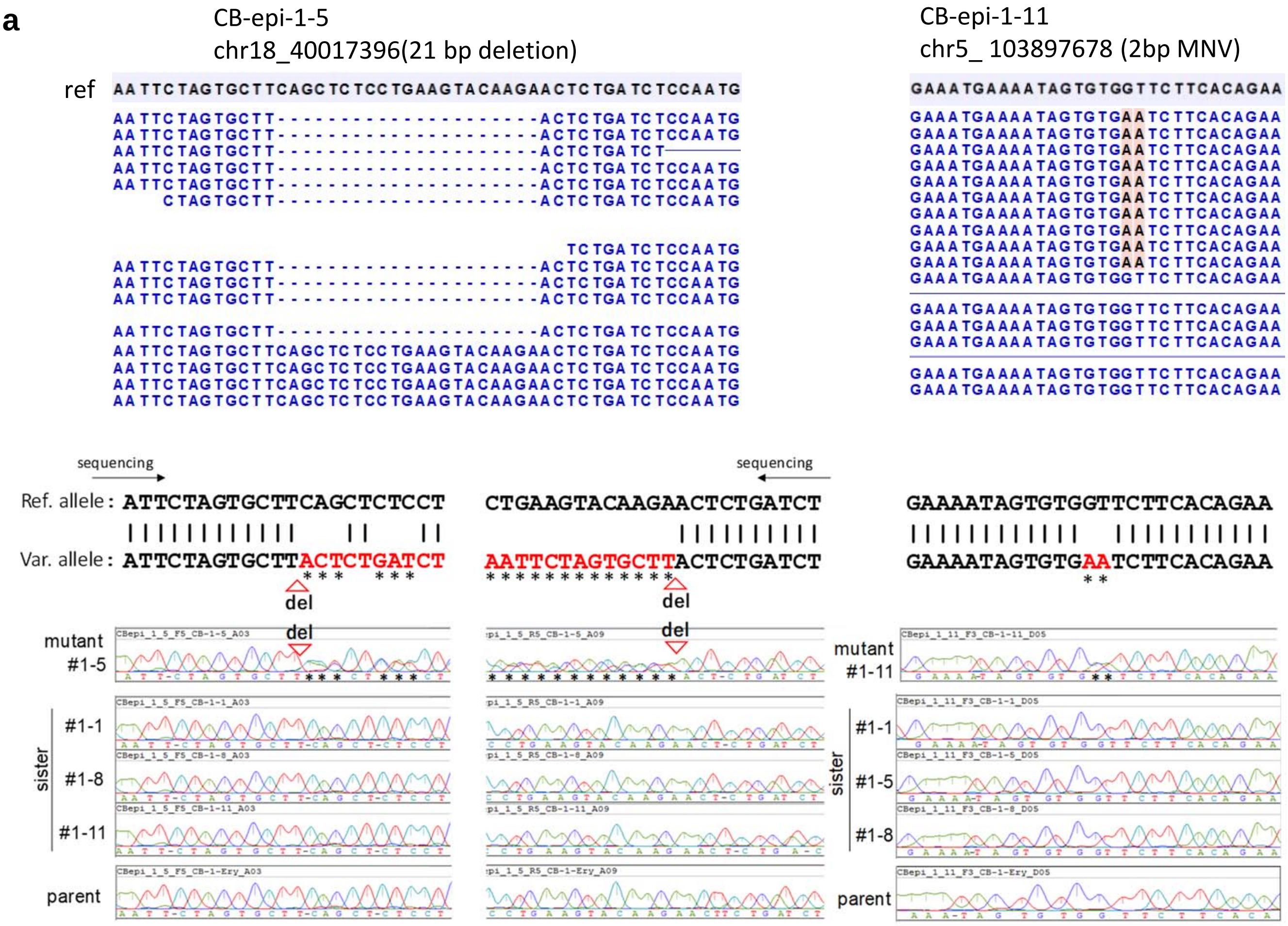

b

Name	Position	Type	Reference Allele	Variant Allele	Length	Sanger Seq	Primer F	Primer R
CB-epi-1-1	chr4_66463812	Insertion	-	TATAAATATAG	11	TRUE	ACCTGCCAGCACAGACTAAGA	GCCAATTTAGTGAATTTTACAGAGC
CB-epi-1-1	chr5_114100235	Deletion	TT	-	2	TRUE	TGAGACTAGGATTGCAAACCCTG	CAAATAGGATCATAATGACAGGATCG
CB-epi-1-1	chr11_24541833	Deletion	GAAAAGCAATATTTGG AAATG	-	21	TRUE	GCTGTCAAGAAGATGCAATGG	TTTCTGTGGAAGGGGTTCCT
CB-epi-1-1	chr21_21684443	Deletion	TATAATG	-	7	TRUE	TGAAATAATACCACTGTGCAAACTC	GCATGAGGACTTAAATATTTTGCA
CB-epi-1-5	chr6_102719633	Deletion	T	-	1	TRUE	CTCTTCTCTTGAACCAGACATTCA	AAGTCACAGCCTCAACACTGTC
CB-epi-1-5	chr8_139417404	MNV	GT	TC	2	TRUE	TGCAGCTATAGCCTCACTAAATGT	CATTGTGCACTTGTACCCTAAAAC
CB-epi-1-5	chr9_106231911	MNV	GC	AA	2	FALSE	ATCCCTGCTCGCTTTTGGT	AGCATGCAGGCAACAAACAG
CB-epi-1-5	chr12_78045859	Deletion	T	-	1	TRUE	TAGTGGTGGGGATGTGATGA	ATGATATTGGGAATTTGCTATTTGTT
CB-epi-1-5	chr18_40017396	Deletion	CAGCTCTCCTGAAGTA CAAGA	-	21	TRUE	AGCCTAGGAAGAACAGGCATC	CTCCAAGTTGTTTTGCAAGGA
CB-epi-1-8	chr1_62742449	Insertion	-	AAAAAAAAAA	10	PCR falied	GGCTGAGGCGGGAGAATC	TCACTTTCCATTATGGGGCTTC
CB-epi-1-8	chr1_8894727	Deletion	CCTCCCGAGTAGCTGG ACTACAGGCATGTGCC	-	32	TRUE	AAGAATCTTTTGGCTGTAGTTGTAAT ATC	CCTGGCCAAGATGGTGAAC
CB-epi-1-8	chr4_86789051	Insertion	-	ATATAT	6	PCR falied	TCCTTTAAAGGACTTTTAGATCATCT AGAT	TTCTTAGGACTTCCTGAGGCTG
CB-epi-1-8	chr5_40346530	Deletion	ATTT	-	4	TRUE	TAGTTTTATTAGGATAGTGAGACTAT GGG	CTGGGTCAACCACACTATCAA
CB-epi-1-8	chr6_2191576	Deletion	CCAGCCCAGGC	-	11	TRUE	ACACTCCCGACCCGCTAT	GCTGCAGATGTGTCTGGGA
CB-epi-1-8	chr7_17000619	Deletion	ATATAAA	-	7	PCR falied	AGAATCTCTTTTTGGAAATGTCTGA	GCAAATAATATATAATACAAAAATGTA AGC
CB-epi-1-8	chr9_127459203	Deletion	TTGGGA	-	6	TRUE	GTACCACCATGCCTGGATAAT	AGCCAGTTCTTTGGAAGGTG
CB-epi-1-8	chr15_88388900	Deletion	CAGGTGGC	-	8	TRUE	AATGTCCCATCCACTCTCCA	TTCACATGTGTGAAGAGGGGTA
CB-epi-1-11	chr1_181321651	Deletion	C	-	1	TRUE	TGGTAGGTTTTAAGAATTGATGGAG	GACACTAACAAGTGGCAGAGGA
CB-epi-1-11	chr2_57396080	Deletion	ATAC	-	4	TRUE	GCTGGAAAGAATAATTTGAGGC	TGCAAAGGAATAAGAGAGGATG
CB-epi-1-11	chr5_103897678	MNV	GT	AA	2	TRUE	CAGTTTATAAAACCATAGTGGGAGTT	ACTGTGAGCTTAAGACTAAGTGAAGG
CB-epi-1-11	chr5_146510587	Insertion	-	T	1	TRUE	CCACTGGAAATCTCTCGCTTAC	AGAGAGGGGCTTCAAGATGACT
CB-epi-1-11	chr10_109055536	MNV	CG	TA	2	TRUE	CTGCAATGAGATACCACCTCAC	CCTTTGGGCAAATACCCAG
CB-epi-1-11	chr10_11246742	Insertion	-	CTCCTCCTCCCCGTC	15	TRUE	AGCTGCTGGAAACCCCTT	ACTTTCCTTTATCTCAGCAACAATAG

Supplementary Fig. 3 Validation of INDELs by Sanger sequencing. (**a**) Two INDELs detected in CB-epi-iPSCs are shown as representative cases. Sanger sequencing was performed with both a forward and reverse primer to obtain conclusive results for the two INDELs which revealed a deletion (21bp) and MNV (2bp). Comparison between an iPSC clone harboring an INDEL and its sister clones established from an identical parental erythroblast fraction confirming that both INDELs are clone specific. (**b**) Primers used for the PCR and Sanger sequencing.

Supplementary Figure 4

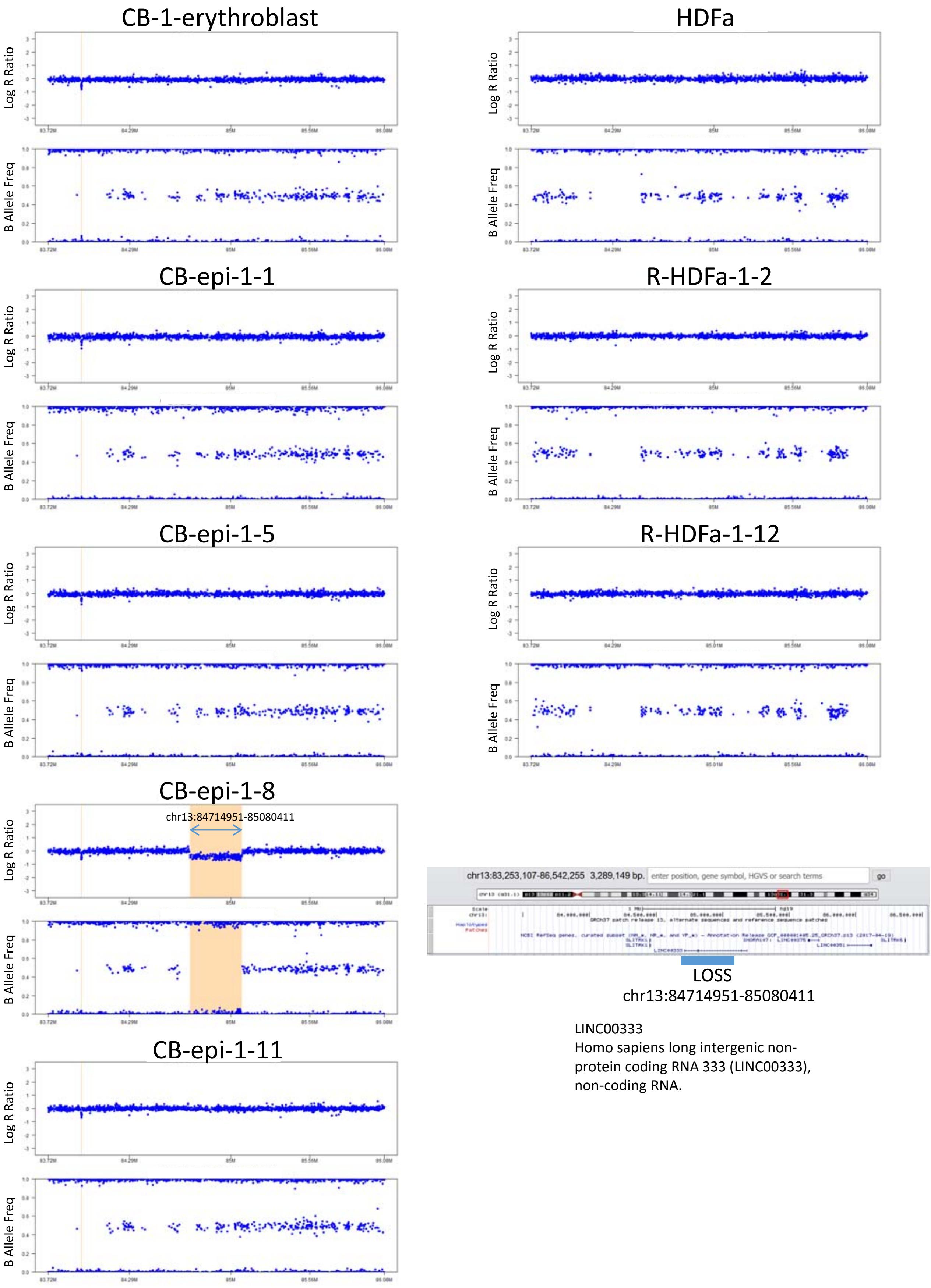

Supplementary Fig. 4 CNV detected by SNP array. An LOH of approximately 365 kbp was identified in chromosome 13 of CB-epi-1-8 iPSC. Log R ratio and B allele frequency plots of chromosome 13 for each iPSC line and their parental cells are shown.

Supplementary Figure 5

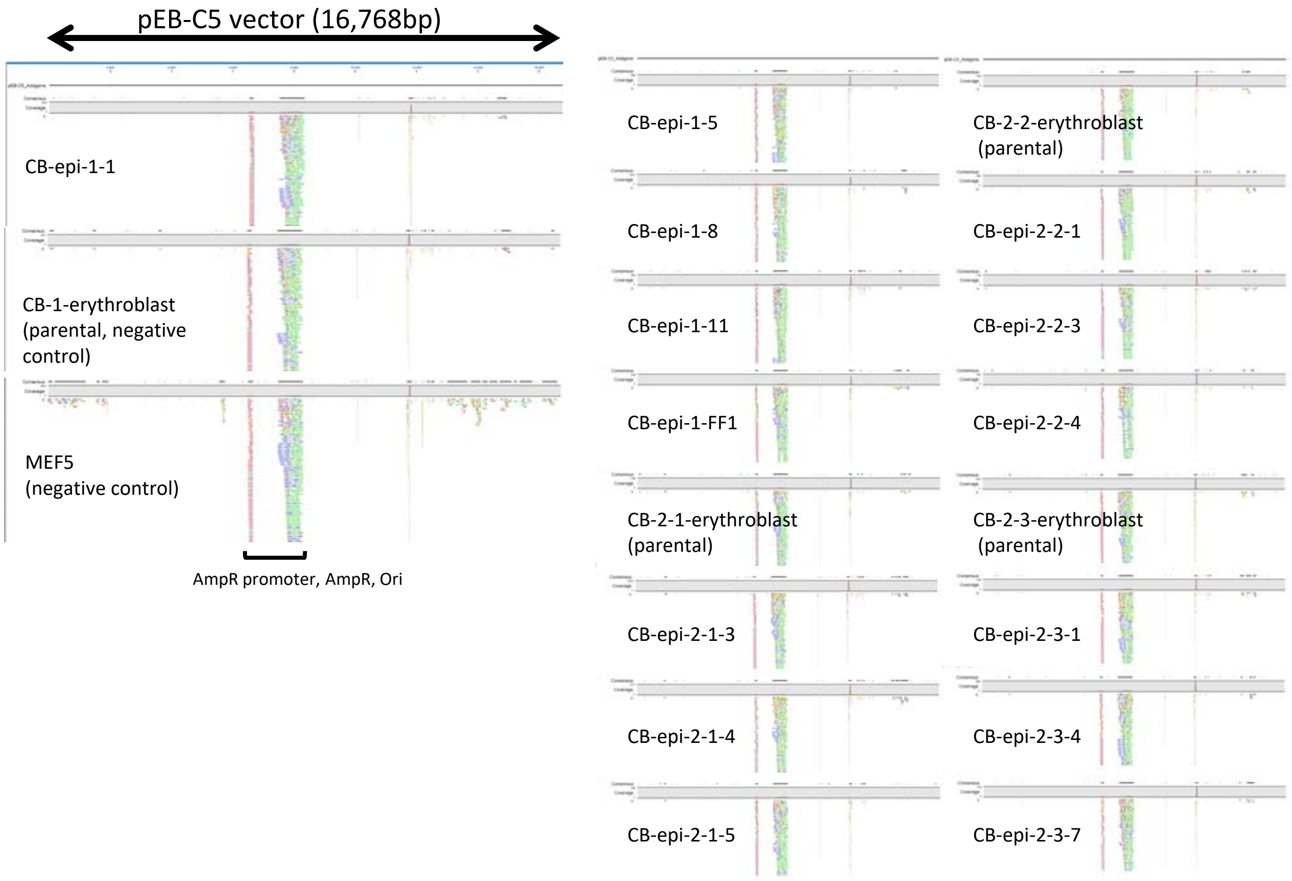

Supplementary Fig. 5 WGS verification of the integration-free status of erythroblast-derived human iPSCs. WGS read mapping with CLC Genomics Workbench to the pEB-C5 vector used for iPSC generation. Integration sites were not detected in iPSC genomes. Note that the mapped sequences, AmpR promoter, AmpR and Ori are also contained in the plasmid used during the preparing of the WGS library.

Supplementary Figure 6

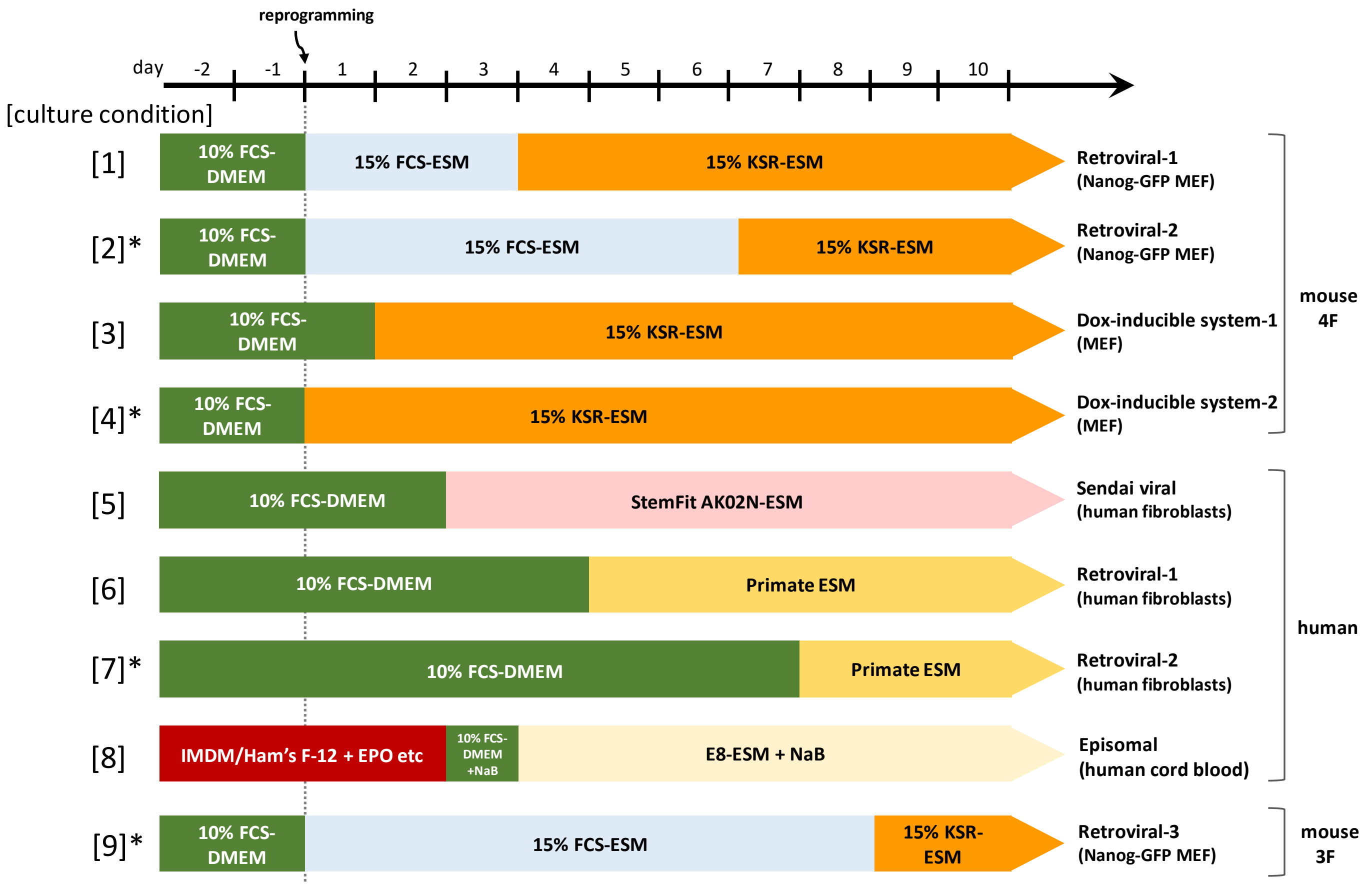

*Improved culture conditions from the original conditions, [2] and [9] from [1] , [4] from [3] and [7] from [6] , to minimize the effects of medium replacement on genome reprogramming, which were used for examining iPSC generation frequency, cell cycle analysis and Western blot.

references
culture condition [1] [2,3]
culture condition [3] [4,5]
culture condition [5] [6]
culture condition [6] [7]
culture condition [8] modified from the original condition [1,8]

Supplementary Fig. 6 Culture conditions used for iPSC generation. Five types of iPSC generation were conducted in this study, two for mouse iPSCs (retroviral and Dox-inducible) and three for human iPSCs (Sendai viral, retroviral and episomal). In addition, a total of nine different culture conditions were employed. Improvements and modifications were made to some of these previously established culture protocols to minimize the effects of medium replacement on iPSC generation (asterisks).

Supplementary Figure 7

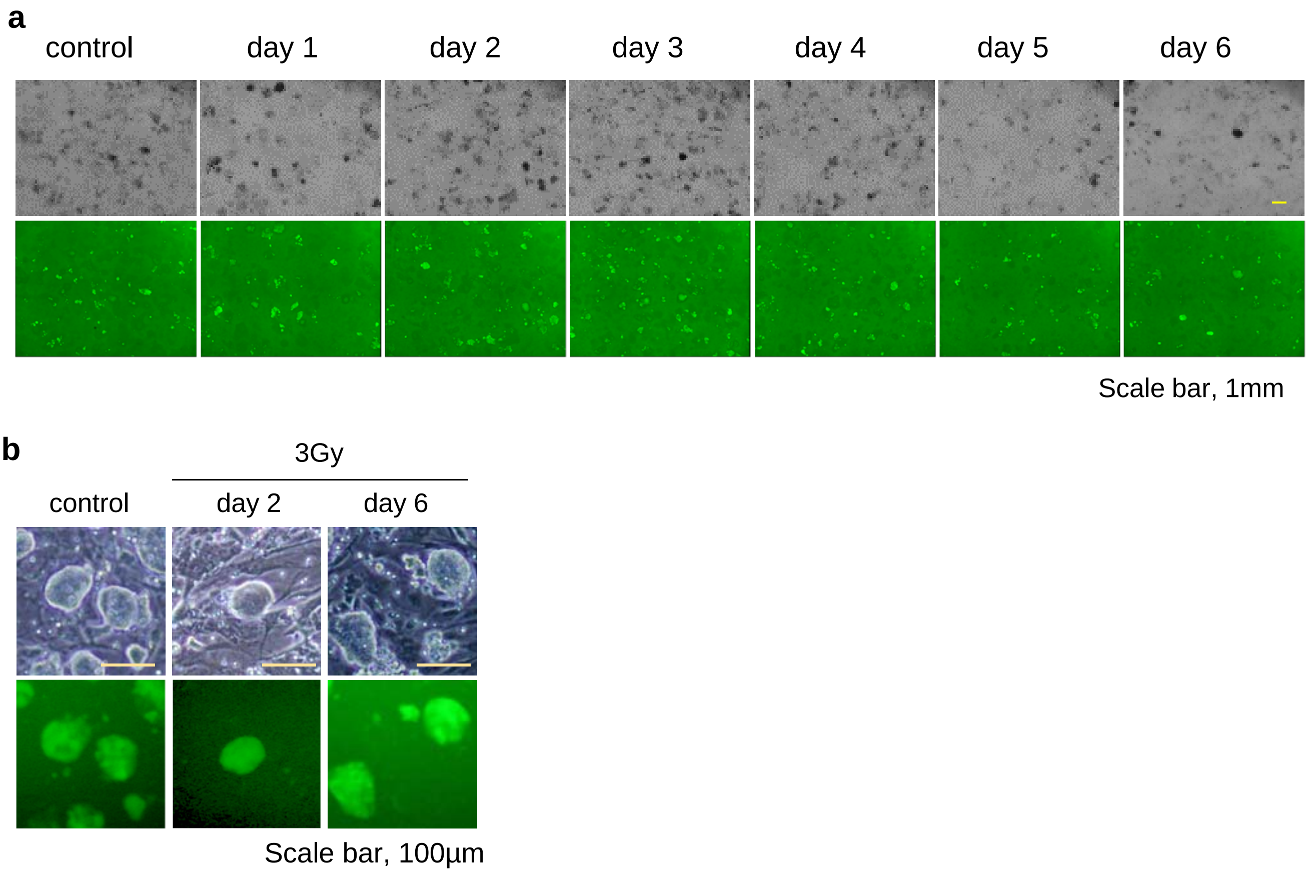

Supplementary Fig. 7 iPSC colony responses to irradiation. (**a**) Representative images of culture dishes at day 14, which were irradiated on days 1, 2, 3, 4, 5 or 6, are shown (Fig. 3a, upper). A retroviral iPSC generation system was employed using Nanog-GFP MEFs and GFP-positive colonies were counted. GFP signals are indicated in the lower panel. (**b**) The cell morphology and GFP expression profile of iPSC lines established after 3 Gy exposure on day 2 or day 6 are shown. These were indistinguishable from the control iPSC clones generated without IR.

Supplementary Figure 8

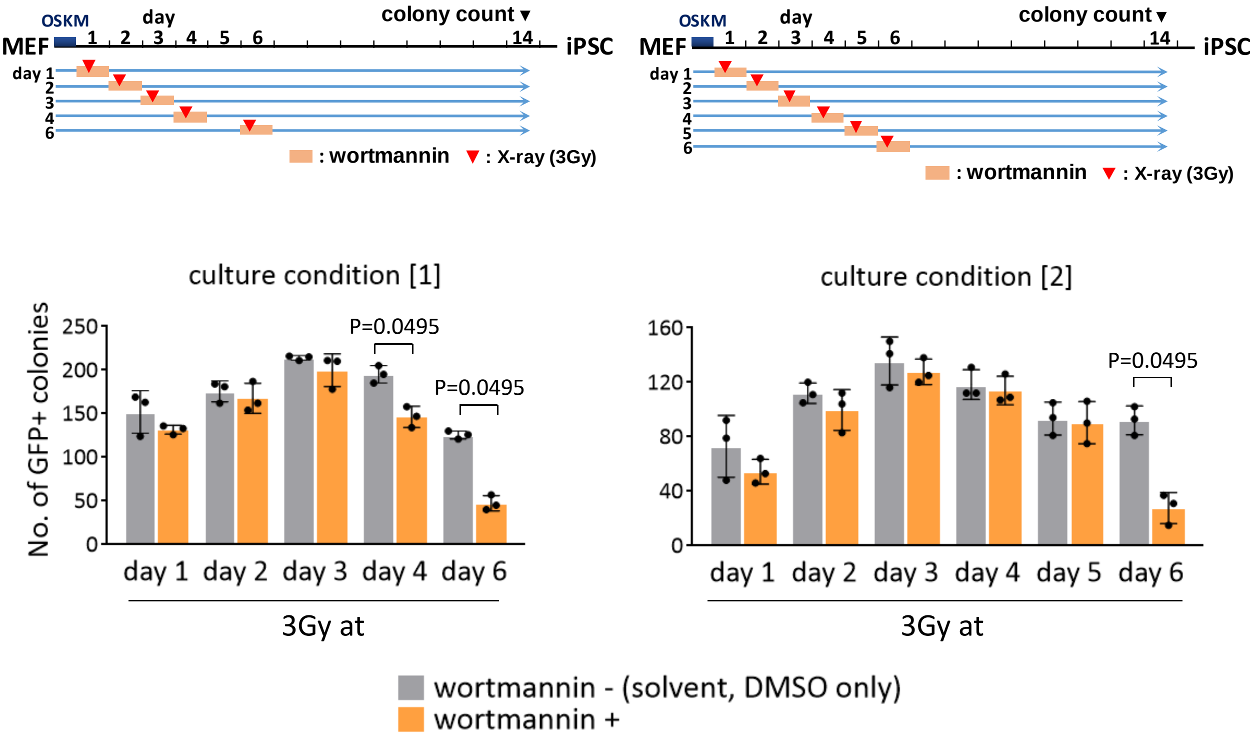

Supplementary Fig. 8 Effects of the checkpoint inhibitor wortmannin on the iPSC generation frequency following irradiation. Left: MEFs infected with OSKM were exposed to 3Gy IR on days 1-4 and 6 post-infection (Supplementary Fig. 6 culture condition [1]). Right: MEFs infected with OSKM were exposed to 3 Gy IR at days 1-6 post-infection (Supplementary Fig. 6 culture condition [2]). Wortmannin was added 15 min prior to irradiation followed by a 23 h, 45 min culture (i.e. 24 h under wortmannin in total). GFP+ colonies were counted on day 14. Error bars show the SD of the mean (n=3 independent dishes). For statistical analysis, two-sided Mann-Whitney U test was employed. Source data are provided as a Source Data file.

Supplementary Figure 9

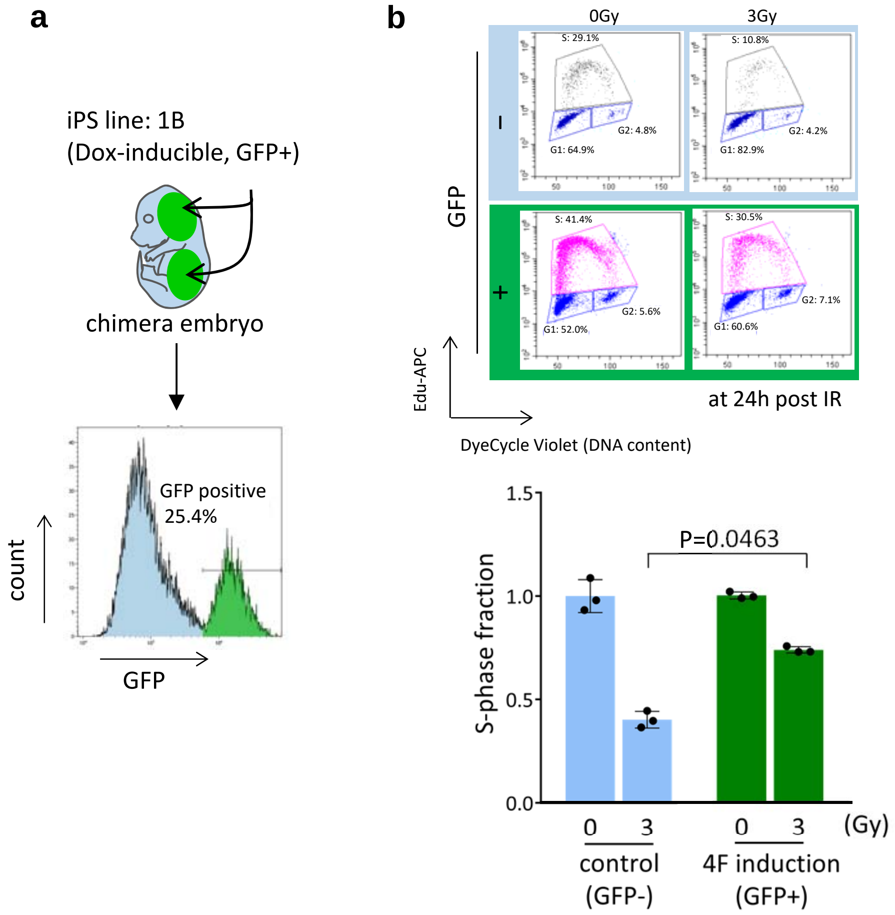

Supplementary Fig. 9 Cell cycle analysis using a Dox-inducible system. (**a**) MEFs derived from a chimeric mouse embryo generated with the 1B line, in which a Dox inducible system for Yamanaka 4 factor expression with the GFP gene is involved, were used to generate secondary iPSCs via Dox induction [4]. The chimeric MEFs were cultured in a dish and then treated with doxycycline. (**b**) Cell cycle analysis with EdU and discrimination of GFP-positive (4F were induced) from GFP-negative (control) cells were performed at 24 h post 3 Gy-irradiation, or in control cells without IR (0 Gy), on day 3. Error bars indicate the SD of the mean (n=3 independent dishes). For statistical analysis, two-sided Mann-Whitney U test was employed. Results are representative of 2 independent experiments with 3 replicates per group. Source data are provided as a Source Data file.

Supplementary Figure 10

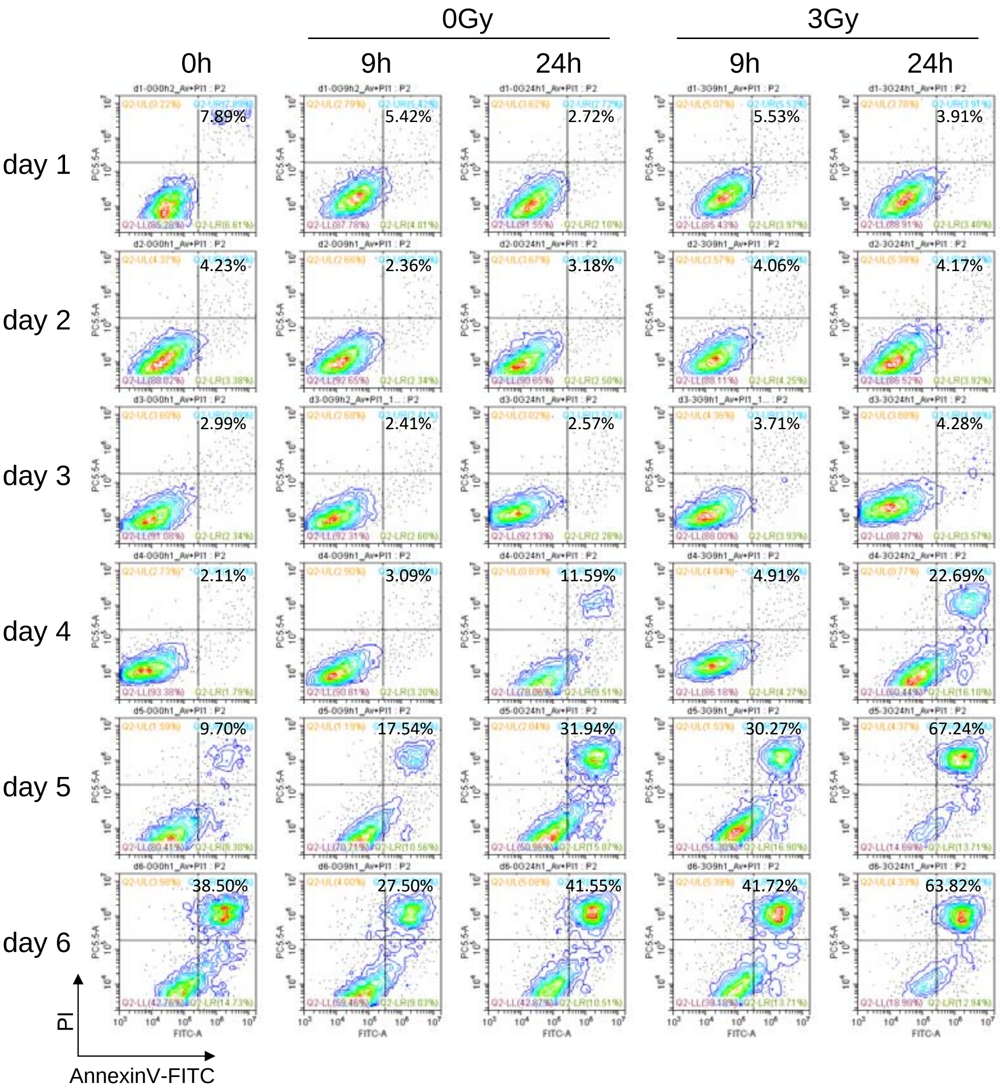

Supplementary Fig. 10 Apoptosis levels at days 1-6 post-infection. 4F-infected MEFs (using a retroviral system) exposed to 3 Gy or without IR (0 Gy) on days 1, 2, 3, 4, 5 or 6 (Fig. 3a, upper) were stained with Annexin V and PI for flow cytometric analysis at 9 h and 24 h post-irradiation. Over 100,000 cells were analyzed for each sample, and the size of the late apoptotic fraction is indicated (upper, right). Analyses of three independent culture dishes and representative data are shown.

Supplementary Figure 11

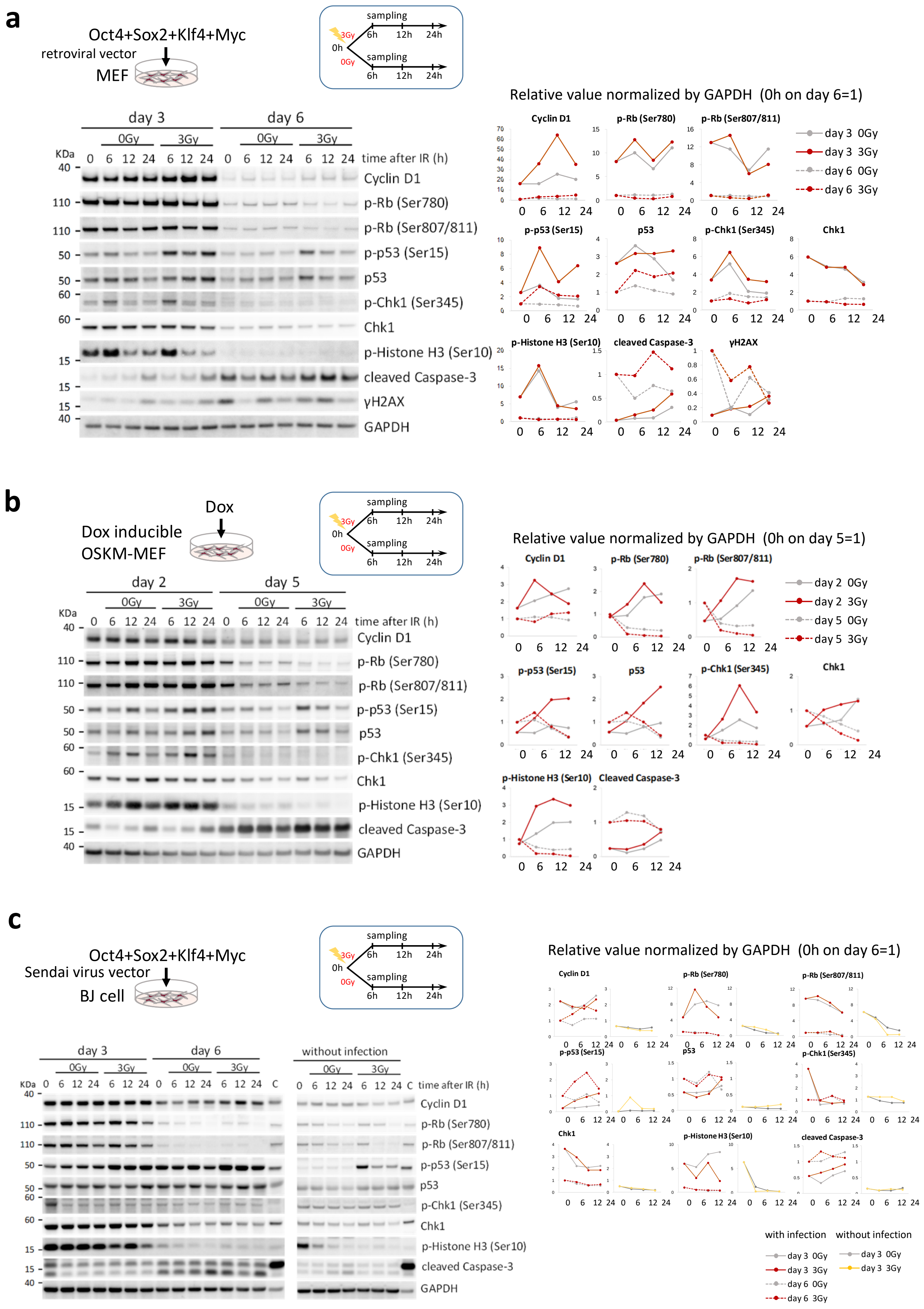

Supplementary Fig. 11 Western blot analysis of cell cycle-related proteins. Western blot analysis was conducted using whole cell lysates prepared at 6, 12 and 24 h after 3 Gy IR or without IR (0 Gy) on day 3 and day 6 (or on day 2 and day 5). The signals were normalized using GAPDH. (**a**) Mouse retroviral system and culture condition [1] (Supplementary Fig. 6). (**b**) Mouse Dox-inducible iPSC generation system. We conducted analysis on day 2 and day 5 in this case because iPSC generation with the Dox-inducible system occurs earlier than that in the retroviral system[4]. Culture condition [4] was used. (**c**) Human iPSC generation using a Sendai viral system with BJ cells with culture condition [5]. Non-infected cells were used as the controls. In addition, because western blot analysis of the 'infected' and 'without infection' sample sets were performed separately (different membranes), an internal control was loaded to normalize the exposure time. Source data are provided as a Source Data file.

Supplementary Figure 12

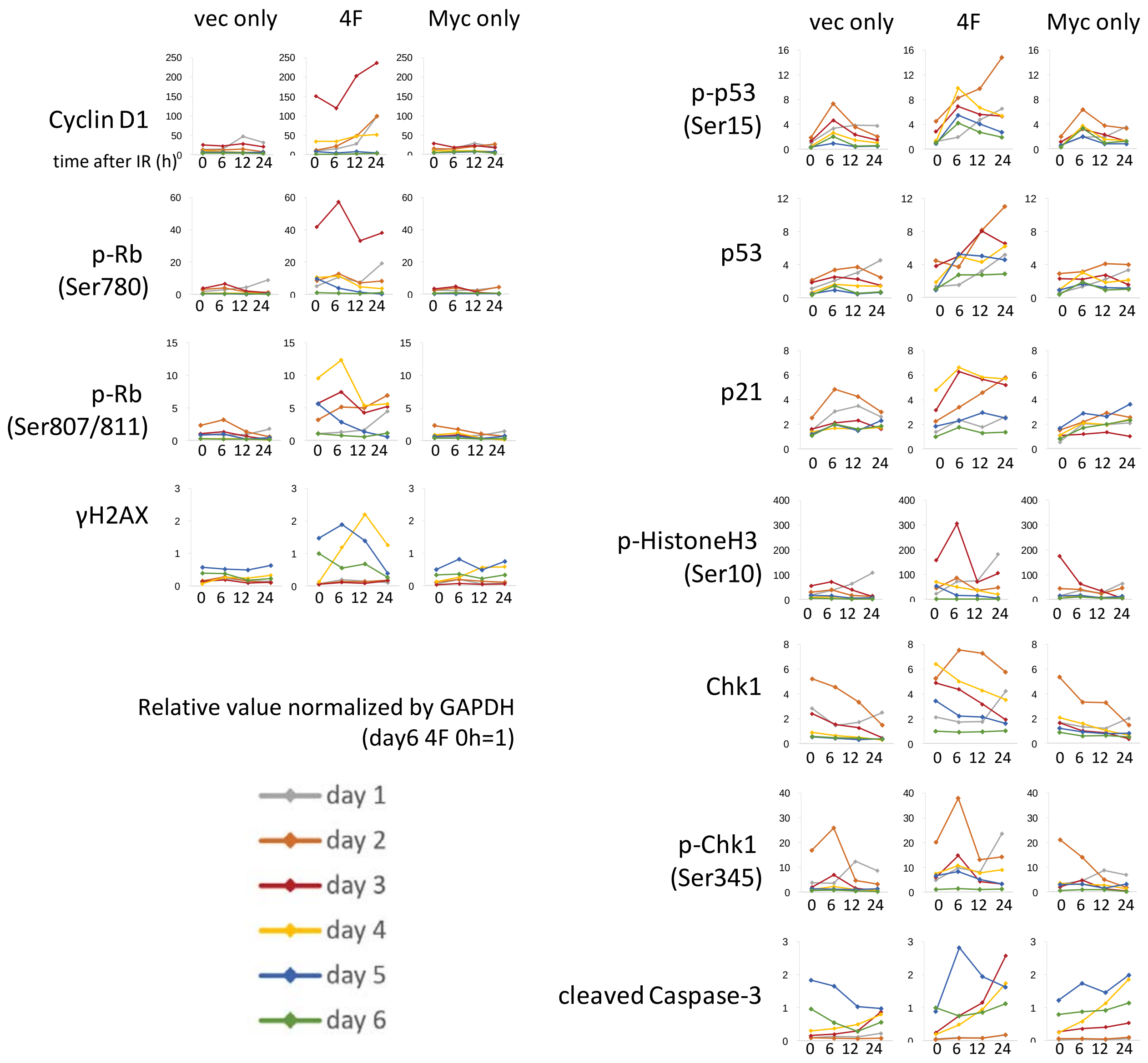

Supplementary Fig. 12 Summary of the immunoblotting of cell cycle-related proteins on days 1-6 using whole cell lysates prepared at 0, 6, 12 and 24 h after 3 Gy irradiation. All signals were normalized to a GAPDH control signal. The background level, which was observed in the non-infected controls, and in the vector only and/or Myc only infections, is indicated by the shaded box.

Supplementary Figure 13

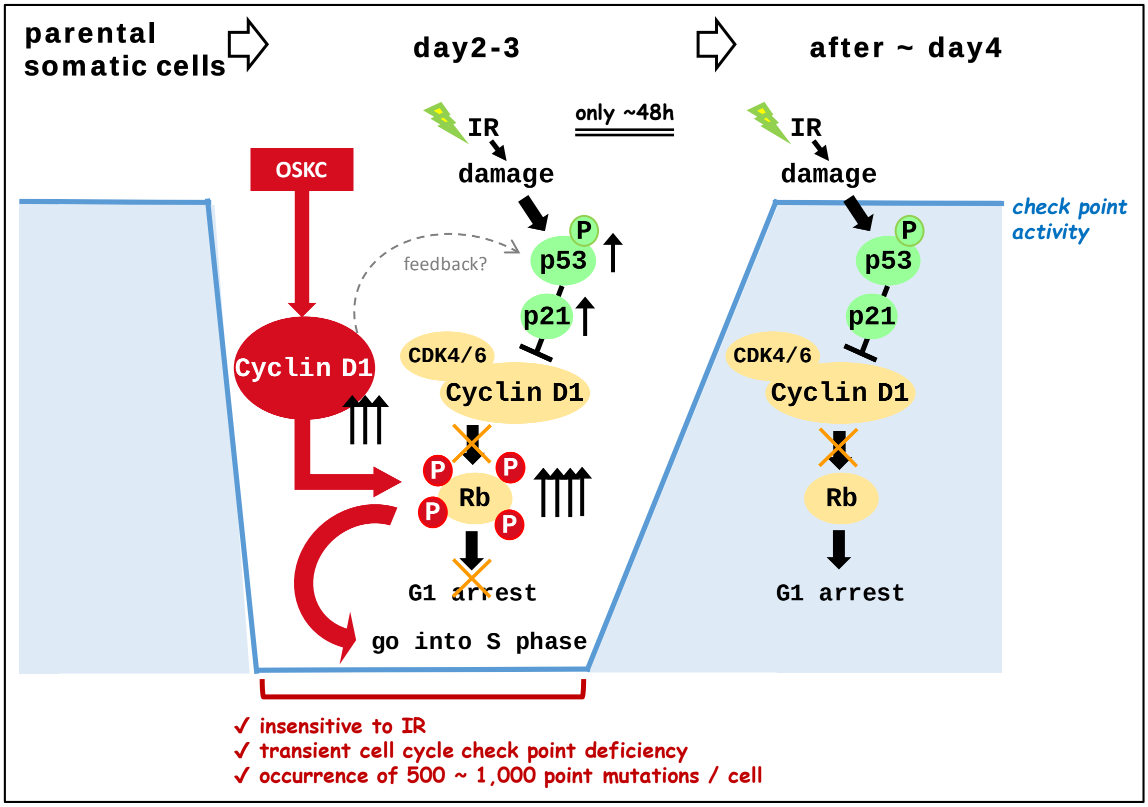

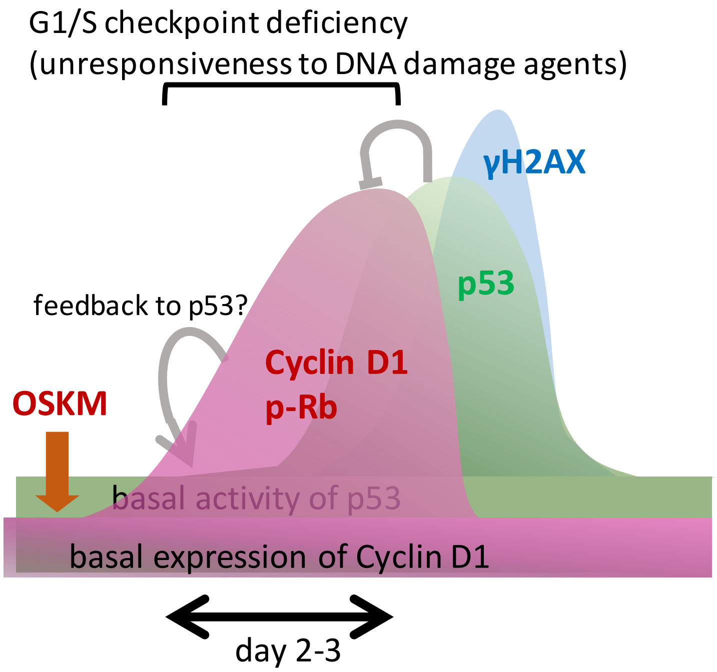

Supplementary Fig. 13 Proposed molecular model for the transient cell cycle checkpoint deficiency at the early stages of iPSC generation. OSKM expression strongly induces cyclin D1 which bypasses p53 control mechanisms leading to the phosphorylation of Rb and an inappropriate G1/S transition. p53 may subsequently be activated by feedback from the enhancement of cyclin D1-Rb phosphorylation, and quickly downregulate the expression of cyclin D1. This mechanism thus causes an attenuation of the cell cycle checkpoint at G1/S in a transient manner. This transient cancer-like phase leads to the accumulation of point mutations and the generation of certain cancer signatures.

Supplementary Figure 14

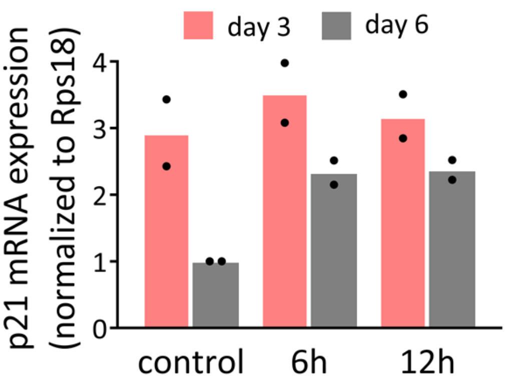

Supplementary Fig. 14 Expression of p21 on days 3 and 6 during mouse iPSC generation using the retroviral system. Real time PCR was performed with total RNA fractions prepared at 6 or 12 h after 3 Gy IR. Controls were not irradiated (n=2 independent iPSC generations for each sampling point).

Supplementary Figure 15

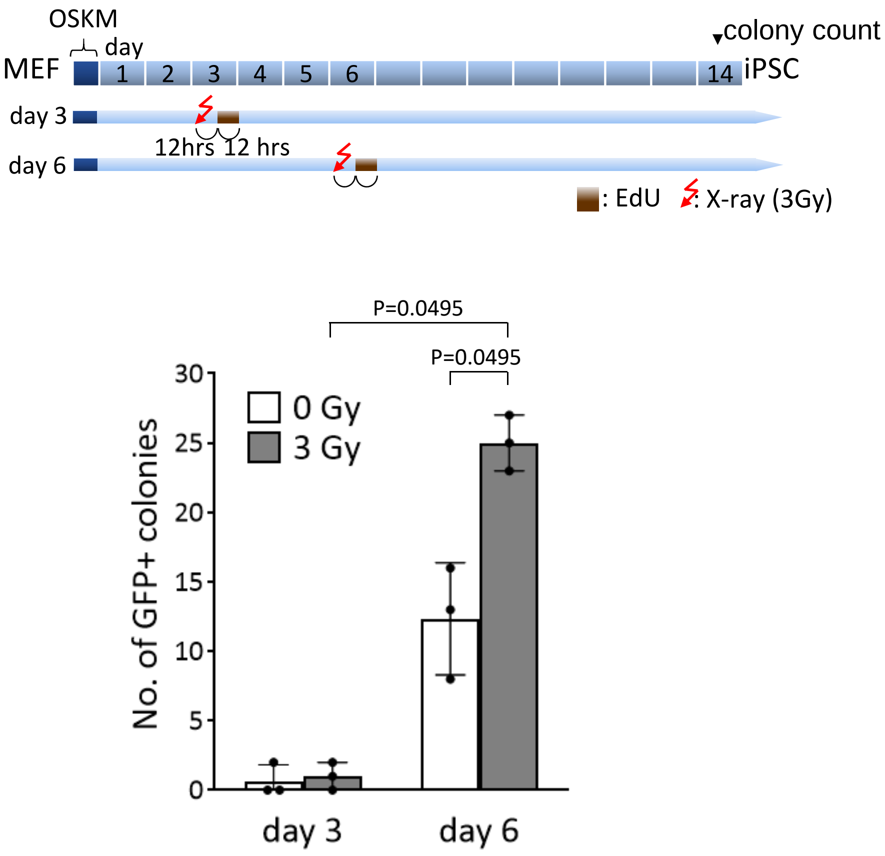

Supplementary Fig. 15 Analysis of cell cycle arrest deficiency using the nucleotide analog EdU. Cells infected with OSKM were exposed to 3 Gy IR on day 3 or day 6 post-infection. EdU was subsequently added at 12 h after IR for a further 12 h. GFP+ colonies were counted on day 14. Error bars show the SD of the mean (n=3 independent dishes). For statistical analysis, two-sided Mann-Whitney U test was employed. Source data are provided as a Source Data file. Results are representative of 2 independent experiments with 3 replicates per group.

Supplementary Figure 16

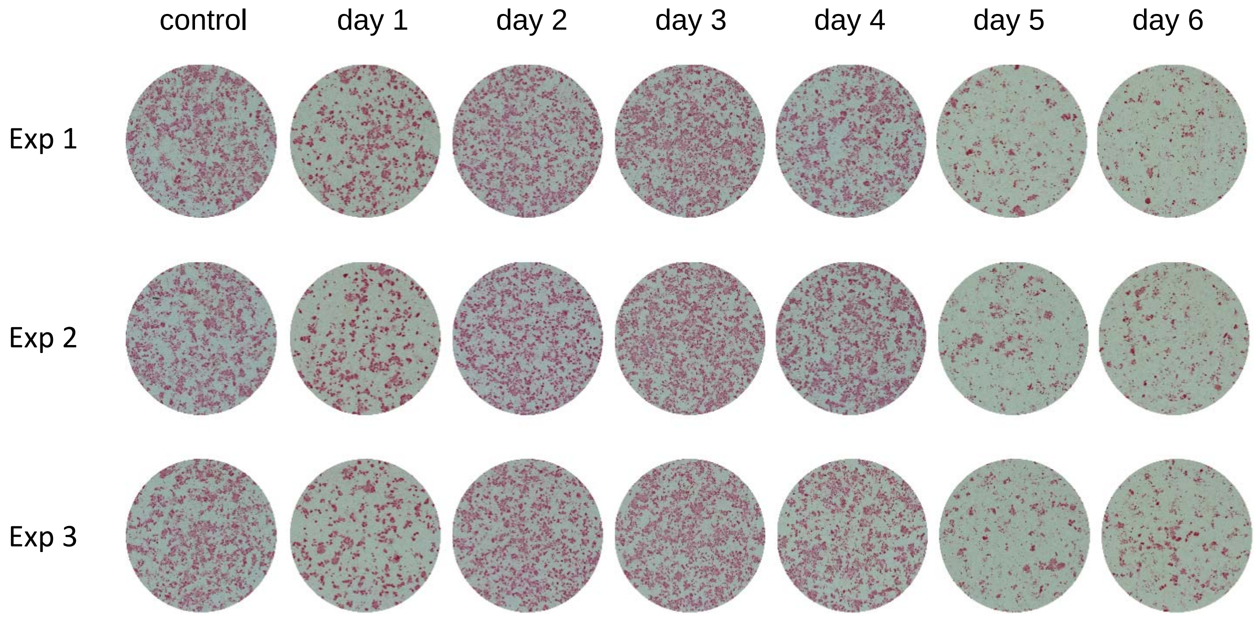

Supplementary Fig. 16 Culture dish images of iPSC generation assay. Mouse Dox-inducible iPSC generation system. Alkaline phosphatase-positive colonies are shown (Supplementary Fig. 6 culture condition [4]).

Supplementary Figure 17

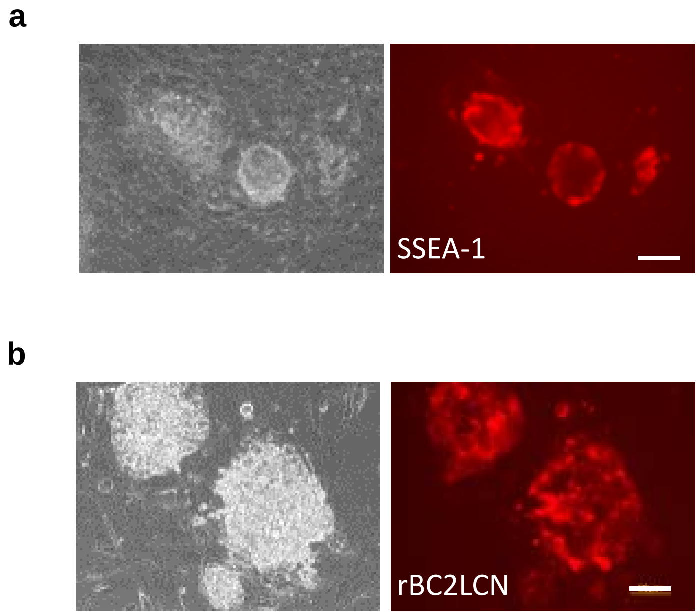

Supplementary Fig. 17 Expression of pluripotent markers. (**a**) SSEA-1 live staining for Dox-induced mouse iPSCs. (**b**) rBC2LCN live staining for human iPSCs generated using the Sendai viral system. Scale bar, 100μm.

Supplementary Figure 18

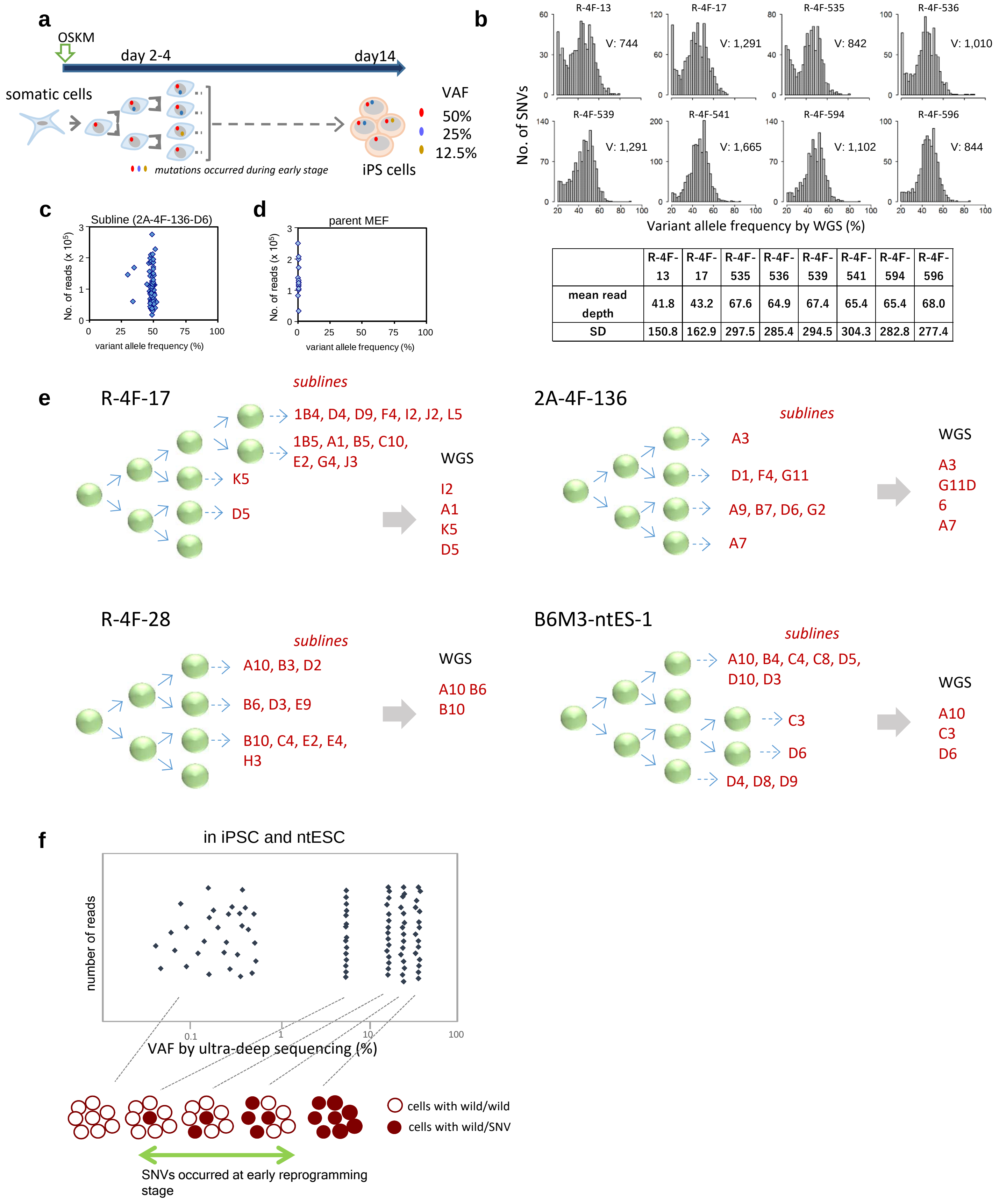

	R-4F-13	R-4F-17	R-4F-535	R-4F-536	R-4F-539	R-4F-541	R-4F-594	R-4F-596
mean read depth	41.8	43.2	67.6	64.9	67.4	65.4	65.4	68.0
SD	150.8	162.9	297.5	285.4	294.5	304.3	282.8	277.4

Supplementary Fig. 18 Comprehensive identification of *de novo* SNVs. (**a**) Relationship between the timing of SNV emergence and their variant allele frequency (VAF) value during iPS generation. A VAF depends on the timing of SNV emergence during iPSC generation. Namely, pre-existing or *de novo* SNVs that emerge in a stem cell which has just been converted from a somatic fibroblast show a 50%VAF, but SNVs that emerge after this show a <50%VAF. (**b**) SNVs identified in 8 independent iPSC clones by WGS using HiseqX. Histograms of the VAFs are shown. V, number of SNVs (20%<= VAF). The mean read depths and standard deviations (SD) are shown (excluding 0 read region). (**c**) Ultra-deep sequencing of subline genomes. Seventy seven randomly chosen SNVs identified in clone 2A-4F-136-D6 were examined. (**d**) We randomly picked 20 SNVs identified in the sublines derived from 2A-4F-136 for the ultra-deep sequencing of their parent MEF genomes (10,000 cells) to verify that they were not pre-existing mutations. (**e**) Classification of the sublines used for comprehensive SNV identification by WGS. A portion of these results have been previously reported [9]. See also Supplementary Data 3. (**f**) Depiction of the relationship between VAF observed by ultra-deep sequencing and mosaicism within a colony.

Supplementary Figure 19

a

sample	dilution ratio (SNV/wild) %	cells	ratio of cells
1	25.0	B6:B10	50:50
2	12.5	B6:B10: 1z-1	25:25:50
3	6.3	B6:B10: 1z-1	12.5:12.5:75.0
4	3.1	B6:B10: 1z-1	6.25:6.25:87.5
5	1.6	B6:B10: 1z-1	3.125:3.125:93.75
6	0	1z-1	100

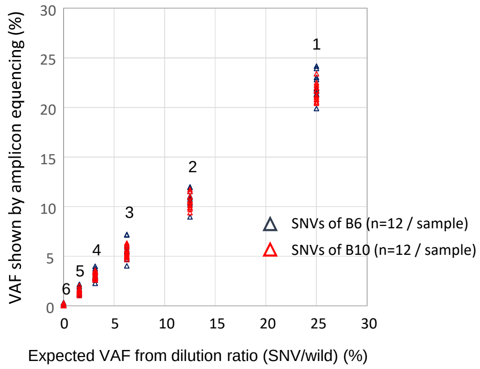

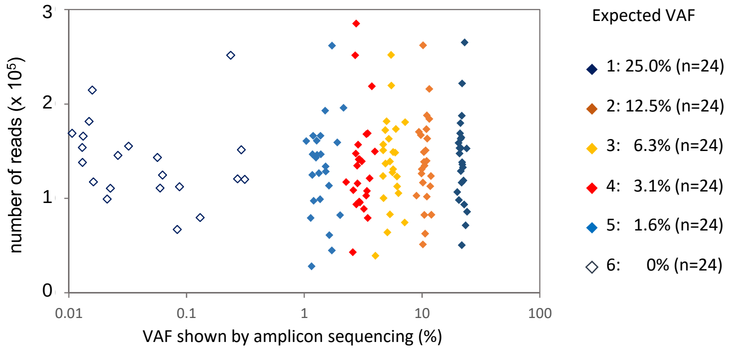

b

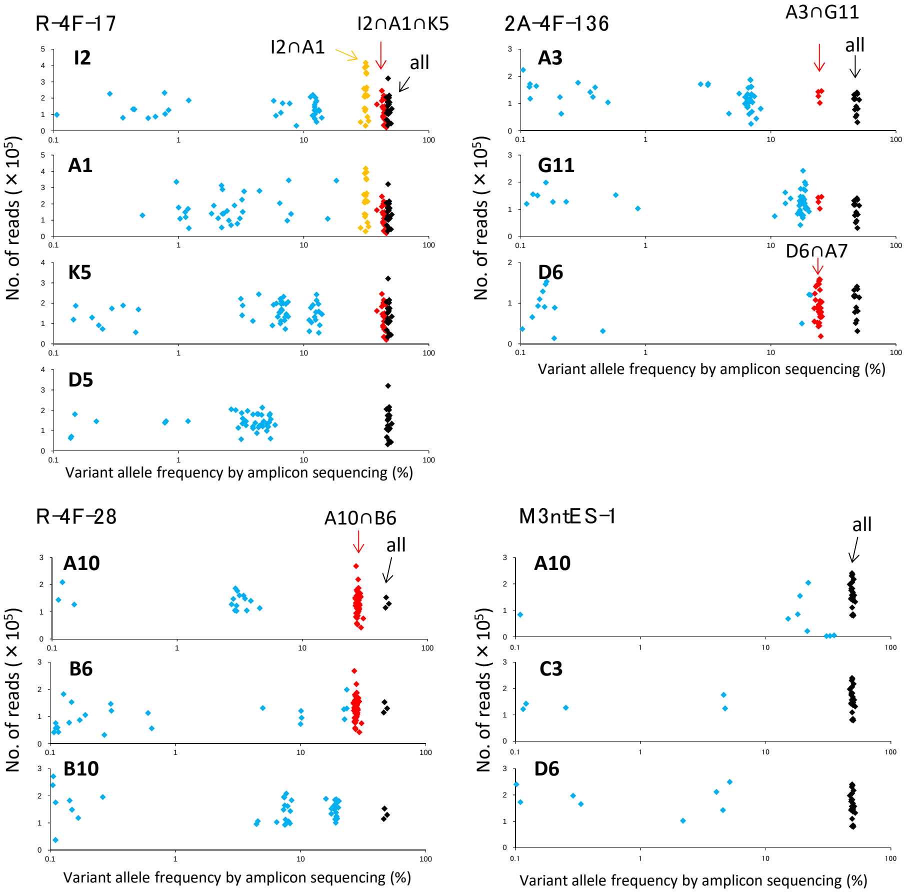

Supplementary Fig. 19 Comprehensive VAF analysis by ultra-deep sequencing. (**a**) Validation of the VAF analysis in this study. We comprehensively identified SNVs, including extremely low frequency mutations, via WGS of single cell-derived cell lines prepared from an iPSC colony, and determined the VAF of each mutation in the parent iPSC genome by amplicon sequencing. We first performed a control experiment using 24 randomly chosen SNVs, 12 identified in the R-4F-28-B6 subline and 12 in the R-4F-28-B10 subline. B6, B10, and 1z-1 (negative control) cells were mixed in various ratios and used in the amplicon sequencing test. Clone 1z-1 is an unrelated iPSC clone which was reported previously[3,9]. (**b**) VAF mapping in the corresponding parental stem cells for the SNVs identified in each subline. The X- and Y-axes indicate the allele frequency and sequence redundancy, respectively.

Supplementary Figure 20

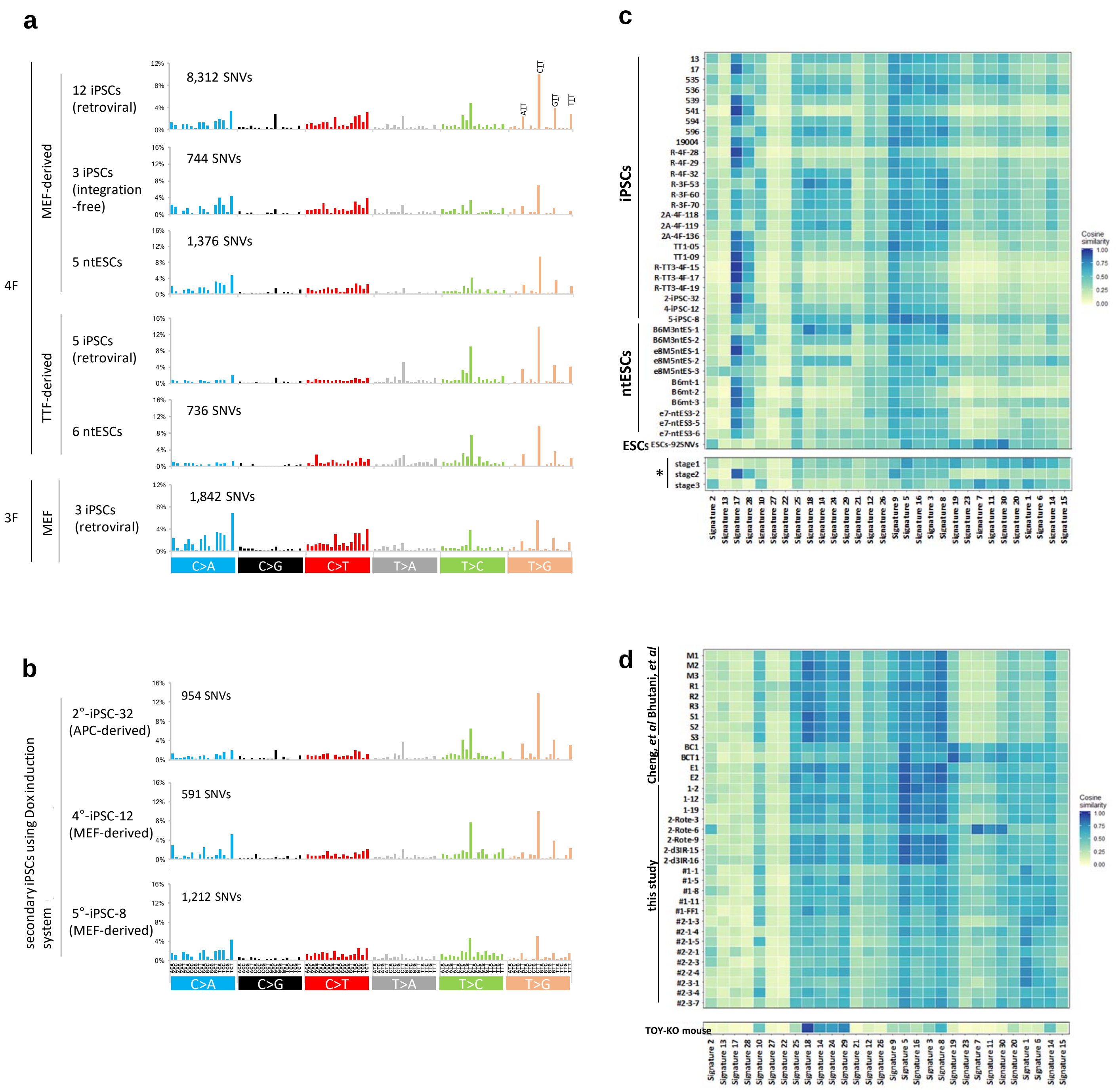

Supplementary Fig. 20 Cancer mutational signatures in reprogrammed pluripotent stem cells. (**a**) 4F (OSKM)-MEF-iPSCs (retroviral): SNVs were identified in five MEF-derived iPSC lines (R-4F-28, R-4F-29, R-4F-32 [9], M5-R-4F-13, 17, 535, 536, 539, 541, 594, 596 and 19004) which were established using retroviral mediated gene transduction. 4F-MEF-iPSCs (integration-free): SNVs were identified in three MEF-derived mouse integration-free iPSC lines (2A-4F-118, 2A-4F-119 and 2A-4F-136) generated using the 2A-plasmid vector system [9]. MEF-ntESCs: SNVs were identified in five MEF-derived ntESC lines (B6M3-ntES-1, B6M3-ntES-2, e8M5-ntES-1, e8M5-ntES-2, and e8M5-ntES-3) established by somatic cell nuclear transfer [10]. 4F-TTF-iPSCs: SNVs were identified in five TTF-derived iPSC lines (R-TT3-4F-15, R-TT3-4F-17, R-TT3-4F-19 [10], TT1-R-4F-05 and TT1-R-4F-09) established by retroviral gene transduction. 4F-TTF-ntESCs: SNVs were identified in six TTF-derived ntESC lines (e7-ntES3-2, e7-ntES3-5, e7-ntES3-6, B6mt-1, B6mt-2 and B6mt-3) established using somatic cell nuclear transfer[10]. 3F (OSK)-MEF-iPSCs: SNVs were identified in three MEF-derived iPSC lines (R-3F-53, R-3F-60 and R-3F-70) established by retroviral gene transduction. (**b**) Raw WGS data (accession no: SRA099420) that had been deposited by the authors in the public database were downloaded and used to identify *de novo* SNVs [11]. (**c**) Similarity between the mutational signatures identified in each mouse iPSC genome and the COSMIC cancer mutational signature calculated using cosine similarity [12]. *See Figure 6a for 'stage 1, 2 and 3'. (**d**) Cosine similarity between the mutational signatures identified in each human iPSC genome and the COSMIC cancer mutational signature.

Supplementary Figure 21

a

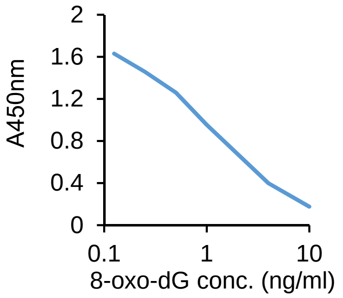

8-oxo-dG (ng/mgDNA)

	control	day3	NAC+ day3	H_2O_2
exp. 1	0.219	0.339	0.218	1.274
exp. 2	0.218	0.322	0.189	1.43
mean	0.22	0.33	0.21	1.35

b

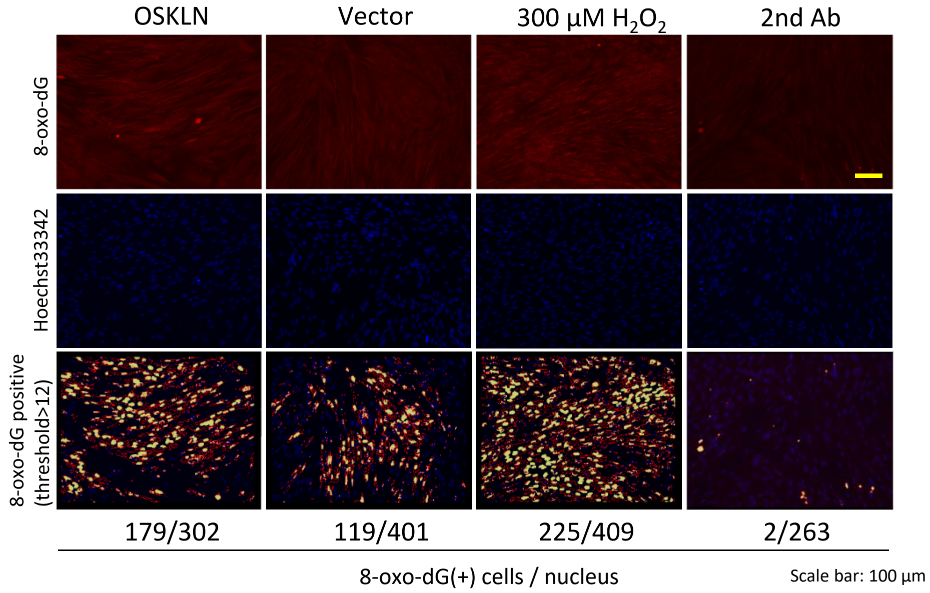

Supplementary Fig. 21 Quantification of 8-oxodG. (**a**) Using the Dox-inducible MEF system, 8-oxo-dG was quantified by ELISA using a mouse anti-8-hydroxy-2'-deoxyguanosine antibody (the N45.1 antibody used for this assay and for immunocytochemistry). Left, standard curve using synthetic 8-oxo-dG substrates. Right, 8-oxo-dG level in the genomic DNA prepared from dox-inducible MEFs. Control MEFs, day 3: MEFs prepared at day 3 post Dox treatment; NAC+day 3, MEFs prepared at day 3 post Dox treatment with 5mM NAC for 24 h on day 3; H_2O_2, MEFs treated with 300μM H_2O_2 for 1 hour at 37° C. (**b**) Quantitative image analysis of 8-oxo-dG positive cells (at day 3 of human iPSC generation). Positive cells (> threshold 12) were selected using the Hybrid cell count program in BZ-analyzer (Keyence) and positive cells that were also Hoechst33342-positive were manually counted. Representative images and whole data are shown.

Supplementary Figure 22

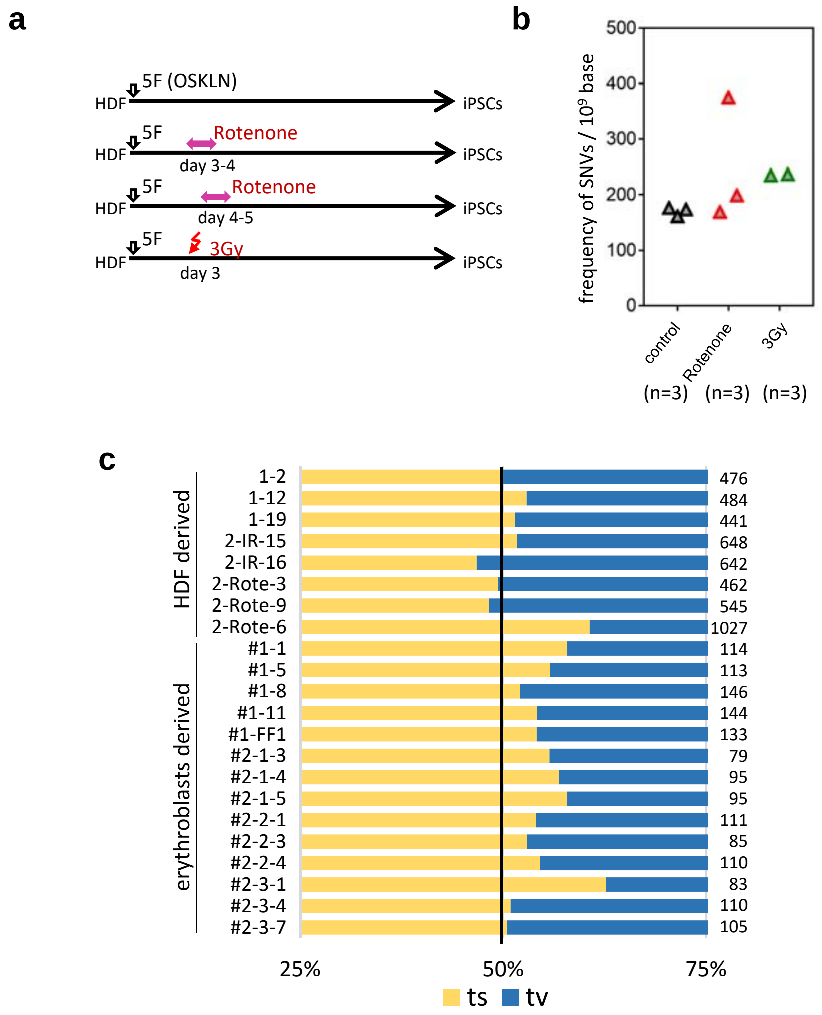

Supplementary Fig. 22 Effects of external stimuli on the point mutation profile in human iPSCs. (**a**) Experimental design for human iPSC generation. (**b**) Effects of ROS-related reagent and IR on the point mutation frequency (n=3 iPSC lines). (**c**) The tv/ts ratio and total number (right side of the panel) of identified SNVs for each clone are shown.

Supplementary Figure 23

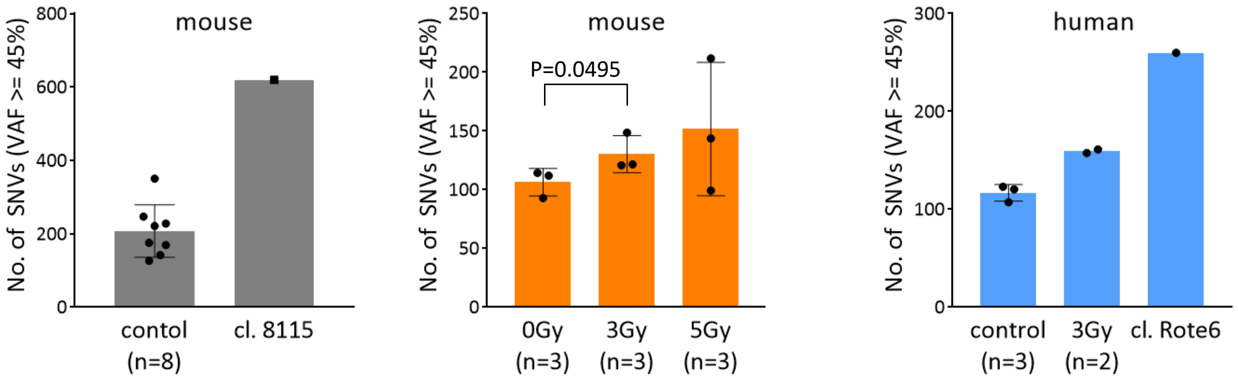

Supplementary Fig. 23 Increase in 50%SNVs by rotenone and ionizing radiation. SNVs (>=45%VAF) are shown to focus on 50%VAF-SNVs (n=1-8 iPSC lines). Error bars show the SD of the mean. For statistical analysis, two-sided Mann-Whitney U test was employed. Source data are provided as a Source Data file.

Supplementary Table 1 Mutations in gene coding regions and their annotations

iPS cell	SNV in coding region												INDEL in coding region				
	number	position	VAF (%)	gene	mutation	codon	amino acid	type	ClinVar*	gnomAD*			number	position	gene	indel	type
										allele count	allele number	allele frequency					
**CB-epi-1-1**	**1**	**chr14_20528851**	**47.6**	**OR4L1**	**T>C**	**GTT>GTC**	**Val>Val**	**synonymous**	**NR**	**2**	**251082**	**7.97E-06**	**0**				
**CB-epi-1-5**	**1**	**chr2_224463804**	**61.3**	**SCG2**	**C>A**	**CGA>CTA**	**Arg>Leu**	**missense**	**NR**	**NR**			**0**				
**CB-epi-1-8**	**1**	**chr12_4763610**	**50.0**	**NDUFA9**	**T>C**	**TAT>CAT**	**Try>His**	**missense**	**NR**	**NR**			**0**				
**CB-epi-1-11**	**0**												**0**				
**CB-epi-1-FF1**	**0**												**0**				
**CB-epi-2-1-3**	**1**	**chr3_186333564**	**42.1**	**AHSG**	**G>T**	**GTG>TTG**	**Val>Leu**	**missense**	**NR**	**NR**			**0**				
**CB-epi-2-1-4**	**2**	**chr11_95712725**	**54.5**	**MAML2**	**G>A**	**TCA>TTA**	**Ser>ter**	**nonsense**	**NR**	**NR**			**0**				
		**chr17_35413915**	**35.9**	**AATF**	**G>A**	**CGC>CAC**	**Arg>His**	**missense**	**NR**	**12**	**282696**	**4.24E-05**					
**CB-epi-2-1-5**	**0**												**1**	**chr22_35726407**	**TOM1**	**insertion (GCAGCTG)**	**truncation**
**CB-epi-2-2-1**	**1**	**chrX_74282206**	**46.4**	**ABCB7**	**A>T**	**CTC>CAC**	**Leu>His**	**missense**	**NR**	**NR**			**0**				
**CB-epi-2-2-3**	**0**												**0**				
**CB-epi-2-2-4**	**0**												**0**				
**CB-epi-2-3-1**	**2**	**chrX_71351954**	**44.0**	**NHSL2**	**T>C**	**AAT>AAC**	**Asn>Asn**	**synonymous**	**NR**	**NR**			**0**				
		**chr17_47300002**	**56.1**	**ABI3**	**C>T**	**TCC>TCT**	**Ser>Ser**	**synonymous**	**NR**	**17**	**281706**	**6.03E-05**					
**CB-epi-2-3-4**	**1**	**chr9_19102644**	**37.5**	**HAUS6**	**G>T**	**AGC>AGA**	**Ser>Arg**	**missense**	**NR**	**NR**			**0**				
**CB-epi-2-3-7**	**1**	**chr2_207460826**	**42.9**	**ADAM23**	**G>T**	**GAT>TAT**	**Asp>Tyr**	**missense**	**NR**	**NR**			**0**				

ClinVar: https://www.ncbi.nlm.nih.gov/clinvar/
gnomAD: https://gnomad.broadinstitute.org/
NR: not reported

Supplementary Table 2 Human iPSC lines used for cancer mutational signature analysis

	gene-delivery systems	iPS cell lines	SNV identification	original data
Dermal fibroblast-derived iPS cells	mRNA, retrovirus and Sendai virus	M1-M2, R1-R3, and S1-S3	by our informatics	Bhutani et al, 2016 [13]
Endothelial progenitor cell-derived iPS cells	retrovirus	S7-RE2 , S7-RE17, S7-RE14	reported*	Rouhani et al, 2016 [14]
Bone marrow cell-derived iPCs	episomal	BC1, BCT1, E1 and E2	by our informatics	Cheng et al, 2012 [15]
R-HDFa	retrovirus	R-HDFa-1-2, R-HDFa-1-12 and R-HDFa-1-19		this study
CB-epi	episomal	CB-epi-1-1, CB-epi-1-5, CB-epi-1-8, CB-epi-1-11, CB-epi-1-FF1, CB-epi-2-1-3, CB-epi-2-1-4, CB-epi-2-1-5, CB-epi-2-2-1, CB-epi-2-2-3, CB-epi-2-2-4, CB-epi-2-3-1, CB-epi-2-3-4 and CB-epi-2-3-7		this study

*Because we found several mutations in the reported SNVs, which are common in these sister lines generated from identical parent cell fraction, we excluded them from our mutational signature analysis.

Supplementary Table 3 Antibodies used for this study

Antibody	Company	Code	experiment	dilution
mouse anti-Cyclin D1 (72-13G)	Santa Cruz Biotechnology	Cat#sc-450	WB	1:1500
mouse anti-Cyclin D1 (G124-326)	BD Biosciences	Cat#554180	WB	1:1000
rabbit anti-phospho-Rb (Ser807/811)	CST	Cat#9308	WB	1:2500
rabbit anti-phospho-Rb (Ser780)	CST	Cat#8180	WB	1:2500
rabbit anti-phospho-p53 (Ser15)	CST	Cat#9284	WB	1:2500
mouse anti-p53 (1C12)	CST	Cat#2524	WB	1:2500
rabbit anti-p53	CST	Cat#9282	WB	1:2000
rabbit anti-phospho-Chk1 (Ser345)	CST	Cat#2348	WB	1:1250
mouse anti-Chk1 (2G1D5)	CST	Cat#2360	WB	1:2500
rabbit anti-phospho-Histone H3 (Ser10)	Merck	Cat#06-570	WB	1:2500
rabbit anti-Cleaved Caspase-3 (Asp175) (5A1E)	CST	Cat#9664	WB	1:2500
rabbit anti-phospho-Histone H2A.X (Ser139)	CST	Cat#9718	WB	1:4000
rabbit anti-G3PDH	Trevigen	Cat#2275-PC-100	WB	1:10000
mouse anti-Pyruvate Dehydrogenase E1-alpha subunit (9H9AF5)	Abcam	Cat#ab110330	WB	1:2000
rabbit anti-phospho-Pyruvate Dehydrogenase E1-alpha subunit (Ser293)(EPR12200)	Abcam	Cat#ab177461	WB	1:2000
rabbit anti-phospho-Pyruvate Dehydrogenase E1-alpha subunit (Ser232)	Merck	Cat#AP1063	WB	1:2500
rabbit anti-phospho-Pyruvate Dehydrogenase E1-alpha subunit (Ser300)	Merck	Cat#AP1064	WB	1:1500
donkey anti-Rabbit IgG, HRP-Linked Whole Ab	GE Healthcare	Cat#NA934	WB	1:10000
sheep anti-Mouse IgG, HRP-Linked Whole Ab	GE Healthcare	Cat#NA931	WB	1:10000
mouse anti-human CD71, PE	BD Biosciences	Cat#555537	FCM	1:5
mouse anti-CD235a (REA175), APC	Miltenyi Biotec	Cat#130-100-262	FCM	1:10
stain alive SSEA-1, mouse anti-Mouse, Dylight 550	Reprocell	Cat#09-0095	ICC	1:100
mouse anti-8-Hydroxy-2'-deoxyguanosine antibody (N45.1)	Abcam	Cat#ab48508	ICC	1:200
rabbit anti-Nanog	Abcam	Cat#ab21624	ICC	1:500
mouse anti-Oct3/4 (C-10)	Santa Cruz Biotechnology	Cat#sc-5279	ICC	1:100
mouse anti-Stage-Specific Embryonic Antigen-4 (MC-813-70)	Merck	Cat#MAB4304	ICC	1:150
mouse anti-TRA-1-60	Merck	Cat#MAB4360	ICC	1:300
mouse anti-TRA-1-81	Merck	Cat#MAB4381	ICC	1:200
mouse anti-Tubulin β 3 (TUBB3)	Biolegend	Cat#801202	ICC	1:500
mouse anti-alpha -Fetoprotein/AFP (189502)	R&D Systems	Cat#MAB1368	ICC	1:200
Rabbit anti-alpha smooth muscle Actin	Abcam	Cat#ab5694	ICC	1:200
rabbit anti-mouse Nanog	Reprocell	Cat#RCAB001P	ICC	1:50
rabbit anti-Oct3/4 (H-134)	Santa Cruz Biotechnology	Cat#sc-9081	ICC	1:100
goat anti-Sox-2 (Y-17)	Santa Cruz Biotechnology	Cat#sc-17320	ICC	1:200
GFP Tag Polyclonal Antibody, Alexa Fluor 488	Thermo Fisher scientific	Cat#A-21311	ICC	1:200
donkey anti-Goat IgG, Alexa Fluor 555	Thermo Fisher scientific	Cat#A-21432	ICC	1:500
goat anti-Rabbiy IgG, Alexa Fluor 488	Thermo Fisher scientific	Cat#A-11034	ICC	1:500
goat anti-Mouse IgG, Alexa Fluor 488	Thermo Fisher scientific	Cat#A-11001	ICC	1:500
goat anti-Mouse IgG, Alexa Fluor 647	Thermo Fisher scientific	Cat#A-21235	ICC	1:500
goat anti-Mouse IgM, Alexa Fluor 555	Thermo Fisher scientific	Cat#A-21422	ICC	1:500

Santa Cruz Biotechnology, Dallas, TX, USA
BD Biosciences, Franklin Lakes, NJ,USA
Cell Signaling Technology (CST), Danvers, MA, USA
Merck, Darmstadt, Germany
Trevigen, Gaithersburg, MD ,USA
GE Healthcare, Chicago, IL ,USA
Stemgent, Beltsville, MD ,USA
Miltenyi Biotec, Bergisch Gladbach, Germany
Reprocell, Yokohama, Japan
Abcam, Cambridge, UK
Biolegend, San Diego, CA, USA
R&D Systems, Minneapolis, MN, USA
Thermo Fisher scientific, Waltham, MA, USA

WB: Western blotting
FCM: Flow cytometry
ICC: Immunocytochemistry

Supplementary References

1. Chou BK, *et al.* Efficient human iPS cell derivation by a non-integrating plasmid from blood cells with unique epigenetic and gene expression signatures. *Cell Res.* **21**, 518-529 (2011).

2. Blelloch R, Venere M, Yen J, Ramalho-Santos M. Generation of induced pluripotent stem cells in the absence of drug selection. *Cell Stem Cell* **1**, 245-247 (2007).

3. Araki R, *et al.* Conversion of ancestral fibroblasts to induced pluripotent stem cells. *Stem Cells* **28**, 213-220 (2009).

4. Woltjen K, *et al.* piggyBac transposition reprograms fibroblasts to induced pluripotent stem cells. *Nature* **458**, 766-770 (2009).

5. Hussein SM, *et al.* Genome-wide characterization of the routes to pluripotency. *Nature* **516**, 198-206 (2014).

6. https://tools.thermofisher.com/content/sfs/manuals/cytotune_ips_2_0_sendai_reprog_kit_man.pdf

7. Yagi T, *et al.* Modeling familial Alzheimer's disease with induced pluripotent stem cells. *Hum. Mol. Genet.* **20**, 4530-4539 (2011).

8. Dowey SN, Huang X, Chou BK, Ye Z, Cheng L. Generation of integration-free human induced pluripotent stem cells from postnatal blood mononuclear cells by plasmid vector expression. *Nat. Protoc.* **7**, 2013-2021 (2012).

9. Sugiura M, *et al.* Induced pluripotent stem cell generation-associated point mutations arise during the initial stages of the conversion of these cells. *Stem Cell Rep.* **2**, 52-63 (2014).

10. Araki R, *et al.* The Number of Point Mutations in Induced Pluripotent Stem Cells and Nuclear Transfer Embryonic Stem Cells Depends on the Method and Somatic Cell Type Used for Their Generation. *Stem Cells* **35**, 1189-1196 (2017).

11. Gao S, *et al.* Unique features of mutations revealed by sequentially reprogrammed induced pluripotent stem cells. *Nat. Commun.* **6**, 6318 (2015).

12. Blokzijl F, Janssen R, van Boxtel R, Cuppen E. MutationalPatterns: comprehensive genome-wide analysis of mutational processes. *Genome Med.* **10**, 33 (2018).

13. Bhutani K, *et al.* Whole-genome mutational burden analysis of three pluripotency induction methods. *Nat. Commun.* **7**, 10536 (2016).

14. Rouhani FJ, *et al.* Mutational History of a Human Cell Lineage from Somatic to Induced Pluripotent Stem Cells. *PLoS Genet* **12**, e1005932 (2016).

15. Cheng L, *et al.* Low incidence of DNA sequence variation in human induced pluripotent stem cells generated by nonintegrating plasmid expression. *Cell Stem Cell* **10**, 337-344 (2012).
