# Supplementary material for: Genetic aberrations in iPSCs are introduced by a transient G1/S cell cycle checkpoint deficiency
Source: Nat Commun. 2020 Jan 10;11:197. doi: 10.1038/s41467-019-13830-x (PMC6954237; doi:10.1038/s41467-019-13830-x)
Supplement: Supplementary file 8 — Description of Additional Supplementary Files [file 41467_2019_13830_MOESM8_ESM.pdf]

Description of Supplementary Data Files

File Name: Supplementary Data 1
Description: Summary of WGS sequencing, SNVs and INDELs in the human iPSCs

File Name: Supplementary Data 2
Description: Details of the INDELs

File Name: Supplementary Data 3
Description: Classification of sublines by Sanger sequencing

File Name: Supplementary Data 4
Description: Summary of WGS sequencing of SNVs in mouse iPSCs and their sublines

File Name: Supplementary Data 5
Description: Primers used for amplicon sequencing
