# Supplementary material for: Genetic aberrations in iPSCs are introduced by a transient G1/S cell cycle checkpoint deficiency
Source: Nat Commun. 2020 Jan 10;11:197. doi: 10.1038/s41467-019-13830-x (PMC6954237; doi:10.1038/s41467-019-13830-x)
Supplement: Supplementary file 9 — Reporting Summary [file 41467_2019_13830_MOESM9_ESM.pdf]

nature research

Corresponding author(s): Ryoko Araki and Masumi Abe

Last updated by author(s): Nov 13, 2019

Reporting Summary

Nature Research wishes to improve the reproducibility of the work that we publish. This form provides structure for consistency and transparency in reporting. For further information on Nature Research policies, see Authors & Referees and the Editorial Policy Checklist.

Statistics

For all statistical analyses, confirm that the following items are present in the figure legend, table legend, main text, or Methods section.

n/a	Confirmed	
☐	☒	The exact sample size (*n*) for each experimental group/condition, given as a discrete number and unit of measurement
☐	☒	A statement on whether measurements were taken from distinct samples or whether the same sample was measured repeatedly
☐	☒	The statistical test(s) used AND whether they are one- or two-sided<br>*Only common tests should be described solely by name; describe more complex techniques in the Methods section.*
☒	☐	A description of all covariates tested
☒	☐	A description of any assumptions or corrections, such as tests of normality and adjustment for multiple comparisons
☐	☒	A full description of the statistical parameters including central tendency (e.g. means) or other basic estimates (e.g. regression coefficient) AND variation (e.g. standard deviation) or associated estimates of uncertainty (e.g. confidence intervals)
☐	☒	For null hypothesis testing, the test statistic (e.g. *F*, *t*, *r*) with confidence intervals, effect sizes, degrees of freedom and *P* value noted<br>*Give P values as exact values whenever suitable.*
☒	☐	For Bayesian analysis, information on the choice of priors and Markov chain Monte Carlo settings
☒	☐	For hierarchical and complex designs, identification of the appropriate level for tests and full reporting of outcomes
☒	☐	Estimates of effect sizes (e.g. Cohen's *d*, Pearson's *r*), indicating how they were calculated

Our web collection on statistics for biologists contains articles on many of the points above.

Software and code

Policy information about availability of computer code

Data collection

-Illumina HiSeq X Ten: RTA 2(Real Time Analysis 2)
-Keyence Biorevo BZ-9000: BZ-II viewer and analyzer
-Beckman Coulter CytoFLEX S: CytExpert 2.0
-GE Healthcare Life Sciences, ImageQuant LAS 4000 mini, Version 1.3

Data analysis

-Burrows-Wheeler Alignment tool (BWA) (version 0.5.9) and basic alignment tool of CLC Genomics Workbench (CLC Bio, Katrinebjerg, Denmark) were used for whole genome sequencing analysis.
-Agilent Feature Extraction software v11.0
-ImageJ 1.52a (NIH image) was used for Western blotting and 8-oxo-dG analysis.
-Ekuseru-Toukei 2012 (Social Survey Research Information) or statistical software R v3.6.1 were used for statistical analysis.

For manuscripts utilizing custom algorithms or software that are central to the research but not yet described in published literature, software must be made available to editors/reviewers. We strongly encourage code deposition in a community repository (e.g. GitHub). See the Nature Research guidelines for submitting code & software for further information.

Data

Policy information about availability of data

All manuscripts must include a data availability statement. This statement should provide the following information, where applicable:
- Accession codes, unique identifiers, or web links for publicly available datasets
- A list of figures that have associated raw data
- A description of any restrictions on data availability

The Raw Illumina sequencing reads generated during the current study are available in the DDBJ Sequence Read Archive (DRA), accession numbers, DRA002912, DRA005034, DRA005296, DRA006232, DRA006233, DRA006234, DRA006457, DRA006458, DRA006622, DRA007325, DRA007336, DRA008453, DRA008459 and DRA009220. The previously generated Raw Illumina sequencing reads analyzed during the current study are also available in the DRA, accession numbers DRA000524, DRA002956 and DRA003544. SNP array data are deposited at the DDBJ, accession number E-GEAD-311. Microarray data are deposited at the Gene

Expression Omnibus (GEO), accession number GSE131648. The source data for Figures 2a, 3a, 4a-c, e, 3c, 7a, 7b, 8d and Supplementary Figures 1c, 8, 9b, 11a-c, 14, 15, 23 are provided in the Source data file. All other data supporting the findings of this study are available from the corresponding author on reasonable request.

Field-specific reporting

Please select the one below that is the best fit for your research. If you are not sure, read the appropriate sections before making your selection.

☒ Life sciences ☐ Behavioural & social sciences ☐ Ecological, evolutionary & environmental sciences

For a reference copy of the document with all sections, see nature.com/documents/nr-reporting-summary-flat.pdf

Life sciences study design

All studies must disclose on these points even when the disclosure is negative.

Sample size

No specific methods were used for sample size estimation. Sample size was determined based on the literature.

Data exclusions

No data were excluded.

Replication

iPS cell generation with cord blood mononuclear cells were performed with 4 biological replications (4 donors). Three or two independent experiments with 3 replicates were performed for each colony formation assay, and the results were confirmed with 4 biologically replication (MEF-retrovirus, MEF-dox-inducible, HDF-retrovirus, HDF-Sendai virus). Gene expression analysis by microarray was performed with 2 (HDFs) or 3 (cord blood) individuals. For each western blotting analysis, 1-3 independent experiments were performed. p21 Real-time PCR was performed with 2 experiments. One - two independent experiments were performed with 3 replicates for cell cycle analyses with FCM, and the results were confirmed with 2 biologically replications (MEF non-viral, human fibroblasts-retrovirus). For 8-oxoG immunostaining, 2 independent experiments were performed.

Randomization

SNVs were randomly chosen for amplicon sequencing analyses.

Blinding

Colony formation assay: blinding was used. Blinding was not necessary in all other cases, because the analyses were objective.

Reporting for specific materials, systems and methods

We require information from authors about some types of materials, experimental systems and methods used in many studies. Here, indicate whether each material, system or method listed is relevant to your study. If you are not sure if a list item applies to your research, read the appropriate section before selecting a response.

Materials & experimental systems

n/a	Involved in the study	
☐	☒	Antibodies
☐	☒	Eukaryotic cell lines
☒	☐	Palaeontology
☐	☒	Animals and other organisms
☒	☐	Human research participants
☒	☐	Clinical data

Methods

n/a	Involved in the study	
☒	☐	ChIP-seq
☐	☒	Flow cytometry
☒	☐	MRI-based neuroimaging

Antibodies

Antibodies used

StainAlive SSEA-1 - DyLight 550 (Stemgent, cat# 09-0095, clone MC-480, isotype Mouse IgM, κ), validation: https://www.stemgent.com/products/show/246
anti-phospho-Histone H2A.X (Ser139) (Cell Signaling Technology, cat# 9718, clone 20E3, Rabbit IgG), validation: https://www.cellsignal.com/products/primary-antibodies/phospho-histone-h2a-x-ser139-20e3-rabbit-mab/9718
anti-phospho-p53 (Ser15) (Cell Signaling Technology, cat# 9284, Rabbit), validation: https://www.cellsignal.com/products/primary-antibodies/phospho-p53-ser15-antibody/9284
anti-p53 (1C12) (Cell Signaling Technology, cat# 2524, isotype Mouse IgG1), validation: https://www.cellsignal.com/products/primary-antibodies/p53-1c12-mouse-mab/2524
anti-phospho-Rb (Ser807/811) (Cell Signaling Technology, cat# 9308, Rabbit), validation: https://www.cellsignal.com/products/primary-antibodies/phospho-rb-ser807-811-antibody/9308
anti-Phospho-Chk1 (Ser345)(133D3) (Cell Signaling Technology, cat# 2348, isotype Rabbit IgG), validation: https://www.cellsignal.com/products/primary-antibodies/phospho-chk1-ser345-133d3-rabbit-mab/2348
anti-phospho-Histone H3 (Ser10) (Merck, cat# 06-570, Rabbit), validation: http://www.merckmillipore.com/JP/ja/product/Anti-phospho-Histone-H3-Ser10-Antibody-Mitosis-Marker,MM_NF-06-570
anti-G3PDH (Trevigen, cat# 2275-PC-100, isotype Rabbit IgG), validation: https://trevigen.com/docs/protocol/protocol_2275-PC-100.pdf
Donkey anti-Rabbit IgG, HRP-Linked Whole Ab (GE Healthcare, cat# NA934), validation: https://www.gelifesciences.co.jp/

catalog/0428.html
Sheep anti-Mouse IgG, HRP-Linked Whole Ab (GE Healthcare, cat# NA931), validation: https://www.gelifesciences.co.jp/catalog/0428.html
anti-8-hydroxyguanosine antibody (Abcam, cat# ab48508, clone N45.1, isotype Mouse IgG κ), validation: https://www.abcam.co.jp/8-hydroxy-2-deoxyguanosine-antibody-n451-ab48508.pdf
Goat anti-mouse IgG (H+L) cross-adsorbed secondary antibody, Alexa Fluor 647 (Molecular Probes/Thermo Fisher Scientific, cat# A-21235, Goat IgG) validation: https://www.thermofisher.com/antibody/product/Goat-anti-Mouse-IgG-H-L-Cross-Adsorbed-Secondary-Antibody-Polyclonal/A-21235
anti-GFP polyclonal antibody-Alexa Fluor 488 (Invitrogen/Thermofisher scientific, cat# A-21311, Rabbit IgG), validation: https://www.thermofisher.com/antibody/product/GFP-Tag-Antibody-Polyclonal/A-21311
mouse anti-Cyclin D1 (72-13G) (Santa Cruz Biotechnology, Cat#sc-450), validation: https://www.scbt.com/scbt/product/cyclin-d1-antibody-72-13g
mouse anti-Cyclin D1 (G124-326) (BD Biosciences Cat#554180), validation: https://www.bdbiosciences.com/eu/applications/research/apoptosis/purified-antibodies/purified-mouse-anti-human-cyclin-d1-g124-326/p/554180
rabbit anti-phospho-Rb (Ser780) (CST Cat#8180), validation: http://media.cellsignal.com/pdf/8180.pdf
rabbit anti-p53 (CST, Cat#9282) Validation: https://www.cellsignal.jp/products/primary-antibodies/p53-antibody/9282
mouse anti-Chk1 (2G1D5) (CST, Cat#2360), validation: https://www.cellsignal.jp/products/primary-antibodies/chk1-2g1d5-mouse-mab/2360
rabbit anti-Cleaved Caspase-3 (Asp175) (5A1E) (CST, Cat#9664), validation: https://www.cellsignal.jp/products/primary-antibodies/cleaved-caspase-3-asp175-5a1e-rabbit-mab/9664
mouse anti-Pyruvate Dehydrogenase E1-alpha subunit (9H9AF5) (Abcam, Cat#ab110330),validation: https://www.abcam.co.jp/pyruvate-dehydrogenase-e1-alpha-subunit-antibody-9h9af5-ab110330.pdf
rabbit anti-phospho-Pyruvate Dehydrogenase E1-alpha subunit (Ser293)(EPR12200) (Abcam, Cat#ab177461), validation: https://www.abcam.co.jp/pyruvate-dehydrogenase-e1-alpha-subunit-phospho-s293-antibody-epr12200-ab177461.pdf
rabbit anti-phospho-Pyruvate Dehydrogenase E1-alpha subunit (Ser232) (Merck, Cat#AP1063), validation: http://www.merckmillipore.com/JP/ja/product/PhosphoDetect-Anti-PDH-E1-pSer232-Rabbit-pAb,EMD_BIO-AP1063#anchor_PDS
rabbit anti-phospho-Pyruvate Dehydrogenase E1-alpha subunit (Ser300) (Merck, Cat#AP1064), validation: http://www.merckmillipore.com/JP/ja/product/PhosphoDetect-Anti-PDH-E1-pSer300-Rabbit-pAb,EMD_BIO-AP1064#anchor_PDS
mouse anti-human CD71, PE (BD Biosciences, Cat#555537), validation: https://www.bdbiosciences.com/us/applications/research/stem-cell-research/mesenchymal-stem-cell-markers-bone-marrow/human/positive-markers/pe-mouse-anti-human-cd71-m-a712/p/555537
mouse anti-CD235a (REA175), APC (Miltenyi Biotec, Cat#130-100-262), validation: https://www.miltenyibiotec.com/JP-en/products/macs-flow-cytometry/antibodies/primary-antibodies/cd235a-glycophorin-a-antibodies-human-rea175-1-11.html
rabbit anti-Nanog (Abcam, Cat#ab21624), validation: https://www.abcam.co.jp/nanog-antibody-chip-grade-ab21624.pdf
mouse anti-Oct3/4 (C-10) (Santa Cruz Biotechnology, Cat#sc-5279), validation: https://datasheets.scbt.com/sc-5279.pdf
mouse anti-Stage-Specific Embryonic Antigen-4 (MC-813-70) (Merck, Cat#MAB4304), validation: http://www.merckmillipore.com/JP/ja/product/Anti-Stage-Specific-Embryonic-Antigen-4-Antibody-clone-MC-813-70,MM_NF-MAB4304#anchor_specialoffer
mouse anti-TRA-1-60 (Merck, Cat#MAB4360), validation: http://www.merckmillipore.com/JP/ja/product/Anti-TRA-1-60-Antibody-clone-TRA-1-60,MM_NF-MAB4360#anchor_specialoffer
mouse anti-TRA-1-81 (Merck, Cat#MAB4381), validation: http://www.merckmillipore.com/JP/ja/product/Anti-TRA-1-81-Antibody-clone-TRA-1-81,MM_NF-MAB4381#anchor_specialoffer
mouse anti-Tubulin β 3 (TUBB3) (Biolegend, Cat#801202), validation: https://www.biolegend.com/
mouse anti-alpha -Fetoprotein/AFP (189502) (R&D Systems, Cat#MAB1368), validation: https://resources.rndsystems.com/pdfs/datasheets/mab1368.pdf
Rabbit anti-alpha smooth muscle Actin (Abcam, Cat#ab5694), validation: https://www.abcam.co.jp/alpha-smooth-muscle-actin-antibody-ab5694.pdf
rabbit anti-mouse Nanog (Reprocell, Cat#RCAB001P), validation: https://reprocell.co.jp/wp-content/docs/products/amna/mNanog_Datasheet_C01MG07(RCAB001P).pdf
rabbit anti-Oct3/4 (H-134) (Santa Cruz Biotechnology, Cat#sc-9081), validation: https://www.scbt.com/p/oct-3-4-antibody-h-134?productCanUrl=oct-3-4-antibody-h-134&_requestid=680773
goat anti-Sox-2 (Y-17) (Santa Cruz Biotechnology, Cat#sc-17320), validation: https://www.scbt.com/p/sox-2-antibody-y-17?productCanUrl=sox-2-antibody-y-17&_requestid=681154
donkey anti-Goat IgG, Alexa Fluor 555 (Thermo Fisher scientific, Cat#A-21432), validation: https://www.thermofisher.com/antibody/product/Donkey-anti-Goat-IgG-H-L-Cross-Adsorbed-Secondary-Antibody-Polyclonal/A-21432
goat anti-Rabbiy IgG, Alexa Fluor 488 (Thermo Fisher scientific, Cat#A-11034), validation: https://www.thermofisher.com/order/genome-database/dataSheetPdf?producttype=antibody&productsubtype=antibody_secondary&productId=A-11034&version=84
goat anti-Mouse IgG, Alexa Fluor 488 (Thermo Fisher scientific, Cat#A-11001), validation: https://www.thermofisher.com/order/genome-database/dataSheetPdf?producttype=antibody&productsubtype=antibody_secondary&productId=A-11001&version=84
goat anti-Mouse IgM, Alexa Fluor 555 (Thermo Fisher scientific, Cat#A-21422), validation: https://www.thermofisher.com/order/genome-database/dataSheetPdf?producttype=antibody&productsubtype=antibody_secondary&productId=A-21422&version=84

Validation

All antibodies were validated by the manufacturers as mentioned above.

Eukaryotic cell lines

Policy information about cell lines

Cell line source(s)

PlatE cells were kindly provided by Dr. Kitamura (University of Tokyo).
BJ human foreskin fibroblasts were purchased from ATCC (CRL-2522) .
Jurkat was obtained from RIKEN-BRC.
CB-MNCs were purchased from PromoCell (C-12901).

HDFs were purchased from Lifeline Cell Technology (FC-7 0024) and Thermo Fisher Scientific (C0135C).
iPS cells and ntES cells were originally established in our labs.

Authentication

Cell lines were not externally authenticated, but BJ human foreskin fibroblasts were authenticated using Short Tandem Repeat (STR) analysis by ATCC.

Mycoplasma contamination

Sampling tests using PCR or whole genome sequencing data revealed that the cell lines used in this study are mycoplasma negative.

Commonly misidentified lines (See ICLAC register)

No commonly misidentified cell lines were used in this study

Animals and other organisms

Policy information about studies involving animals; ARRIVE guidelines recommended for reporting animal research

Laboratory animals

Male Nanog-GFP tg [STOCK Tg(Nanog-GFP, puro)1Yam](RIKEN BRC) mice (Okita et al., 2007) were mated with female C57BL/6J (Japan SLC, Hamamatsu, Japan) mice, and MEFs were then prepared from day 13.5 embryos.
The mouse iPSC line, 1B, was generated using piggyBac vectors containing the Dox-inducible reprogramming factors, Oct4, Sox2, Klf4 and c-Myc from ROSA26 knock-in rtTA-IRES-GFP MEFs (Hussein et al., 2014; Woltjen et al., 2009). To establish secondary iPSCs, we prepared MEFs from a chimeric MEFs (1B x BDF1). Briefly, 1B cells (GFP-positive) were injected into BDF1 embryos (8-cell embryos ~ blastocysts) to prepare chimera embryos, and then MEFs were prepared at day 13.5. The donor cell contribution for each MEF was assessed by GFP expression using flow cytometry. The animals were housed under a controlled lighting condition (daily light 07:00–19:00 hr) and used at 8-12 weeks (female) and 8-40 weeks (male). All mouse experiments were performed in accordance with the relevant guidelines and were approved by the Animal Care and Use Committee of the National Institute of Quantum and Radiological Science and Technology, and University of Yamanashi.

Wild animals

The study did not involve wild animals.

Field-collected samples

The study did not involve samples collected from the field.

Ethics oversight

The ethics committees of the National Institute of Radiological Sciences and University of Yamanashi.

Note that full information on the approval of the study protocol must also be provided in the manuscript.

Flow Cytometry

Plots

Confirm that:

☒ The axis labels state the marker and fluorochrome used (e.g. CD4-FITC).

☒ The axis scales are clearly visible. Include numbers along axes only for bottom left plot of group (a 'group' is an analysis of identical markers).

☒ All plots are contour plots with outliers or pseudocolor plots.

☒ A numerical value for number of cells or percentage (with statistics) is provided.

Methodology

Sample preparation

This information is included in the Methods section page.

Instrument

CytoFLEX S from Beckman Coulter

Software

For CytoFLEX S from Beckman Coulter the software to collect data is CytExpert and to analyze data is Kaluza Analysis software.

Cell population abundance

Not applicable.

Gating strategy

Initially cells were gated by FSC-A vs SSC-A for the exclusion of debris.

☐ Tick this box to confirm that a figure exemplifying the gating strategy is provided in the Supplementary Information.
